# Supplementary material for: How do emergency department staff respond to behaviour that challenges displayed by people living with dementia? A mixed-methods study
Source: BMJ Open. 2023 Aug 4;13(8):e075022. doi: 10.1136/bmjopen-2023-075022 (PMC10407375; doi:10.1136/bmjopen-2023-075022)
Supplement: Supplementary data [file bmjopen-2023-075022supp002.pdf]

Dementia in the ED Survey Questions

Start of Block: Default Question Block

Welcome to 'Managing challenging behaviour related to patients in Dementia in the Emergency Department (ED)' online survey. This survey should take no longer than 5 minutes to complete. Before taking part, please read the information and consent form below, and click on the "I consent" button at the bottom of the page if you understand the information and freely consent to participate in the study.

INFORMATION AND CONSENT FORM

This survey is designed to understand ED staff experiences of challenging behaviour from patients with dementia and what interventions they have found useful. This is being led by Dr Sarah Voss and Dr Laura Goodwin of the University of the West of England, Bristol. It has been approved by the University of the West of England Research Ethics Committee.

The aim of this survey is to discover how staff in an ED department manage challenging behaviour exhibited by people with dementia in order to inform the development of a standardised de-escalation tool. These patients can often feel confused and disorientated in a hospital setting and may not be able to express pain or basic care needs. It is important they receive quality care and are as comfortable as possible. Therefore, we are interested to know why these patients may become agitated and how they are managed effectively by staff to avoid distress. The findings from this survey will be combined with data from interviews with ED staff, and may lead on to the development of an intervention to manage challenging behavior from patients with dementia in the ED.

You have been invited to take part because you are a member of staff working in the Emergency Department at either University Hospitals Bristol NHS Foundation Trust, North Bristol NHS Trust, or Royal United Hospitals Bath NHS Foundation Trust.

Participation will involve answering a series of questions about your experiences of challenging behaviour associated with dementia, whilst working in the ED. Survey participation is entirely voluntary, and there are no consequences of either choosing or not choosing to participate. You can stop being part of the study at any time, without giving a reason, but we will keep information about you that we already have. Your rights to access, change or move your information are limited, as we need to manage your information in specific ways in order for the research to be reliable and accurate. If you withdraw from the study, we will keep the information that we have already obtained. You can find out more about how we use your information by contacting dataprotection@uwe.ac.uk or by following this link:

www.hra.nhs.uk/information-about-patients/

Please read the following Privacy Notice before continuing.

For more information and advice, you may contact Dr Sarah Voss by phone (0117 328 8906) or email (sarah.voss@uwe.ac.uk). Alternatively you can contact Dr Laura Goodwin (laura.goodwin@uwe.ac.uk).

Q2 Do you consent to taking part in this study?

- ○ I consent (1)
- ○ I do not consent (2)

End of Block: Default Question Block

Start of Block: Block 3

Q3 Please select which job title best describes your role:

- ○ Nurse (1)
- ○ Doctor (2)
- ○ Porter (3)
- ○ Security staff (4)
- ○ Receptionist (5)
- ○ Admin staff (6)
- ○ Other (please state) (7) ____

Goodwin L, *et al. BMJ Open* 2024; 13:e075022. doi: 10.1136/bmjopen-2023-075022

Q4 How long have you worked in an Emergency Department?

- Less than a year (1)
- 1-2 years (2)
- 3-5 years (3)
- 6-10 years (4)
- 11-15 years (5)
- 16-20 years (6)
- 21-30 years (7)
- 30 years+ (8)

End of Block: Block 3

Start of Block: Block 1

Q5 Have you ever received staff training in relation to dementia?

- Yes (1)
- No (2)

Skip To: End of Block If Q5 = No

Goodwin L, *et al. BMJ Open* 2024; 13:e075022. doi: 10.1136/bmjopen-2023-075022

Q6 When did you receive this training? *(if you have received more than one lot of training, please indicate when you received the **most recent** training)*

- In the last year (1)
- In the last 2-5 years (2)
- 6-10 years ago (3)
- 10+ years ago (4)

Q7 Did this training involve information about how to manage challenging behaviour associated with dementia?

- Yes (1)
- No (2)

Skip To: End of Block If Q7 = No

Q8 How useful was this information?

- Extremely useful (1)
- Very useful (2)
- Moderately useful (3)
- Slightly useful (4)
- Not at all useful (5)

End of Block: Block 1

Start of Block: Block 4

Goodwin L, *et al. BMJ Open* 2024; 13:e075022. doi: 10.1136/bmjopen-2023-075022

Q9 Have you experienced challenging behaviour from patients with dementia (or suspected dementia) in the ED?

- ◯ Yes (1)
- ◯ No (2)

Skip To: End of Block If Q9 = No

Q10 How frequently would you say that you experience/have to manage challenging behaviour associated with dementia (or suspected dementia) in the ED?

- ◯ Daily (1)
- ◯ Weekly (2)
- ◯ Monthly (3)
- ◯ 2-3 times a year (4)
- ◯ Less than 3 times a year (5)

Q11 How many staff members are usually required to manage instances of challenging behaviour associated with dementia (or suspected dementia) in the ED?

- ◯ 1 (1)
- ◯ 2 (2)
- ◯ 3 (3)
- ◯ more than 3 (4)

Goodwin L, *et al. BMJ Open* 2024; 13:e075022. doi: 10.1136/bmjopen-2023-075022

Q12 Please tick all words which describe your experiences of challenging behaviour associated with dementia (or suspected dementia) in the ED.

- ☐ Verbal aggression (1)
- ☐ Physical assault (2)
- ☐ Agitation (3)
- ☐ Frustration (4)
- ☐ Communication difficulties (5)
- ☐ Use of swear words (6)
- ☐ Physical resistance to care (7)
- ☐ Emotional distress (8)
- ☐ Confusion (9)
- ☐ Wandering (10)
- ☐ Disorientation (11)
- ☐ Other (please state) (12) ________________

Goodwin L, *et al. BMJ Open* 2024; 13:e075022. doi: 10.1136/bmjopen-2023-075022

Q13 How is this behaviour usually managed?

- ☐ Pharmaceutical measures (1)
- ☐ Distraction techniques (2)
- ☐ Use of family members/carers (3)
- ☐ Restraint (4)
- ☐ Moving the patient to a quieter environment (5)
- ☐ Other (please state) (6) ____

Q14 In the last three months, which of the following behaviour management practices have you observed?

- ☐ Pharmaceutical measures (1)
- ☐ Distraction techniques (2)
- ☐ Use of family members/carers (3)
- ☐ Restraint (4)
- ☐ Moving the patient to a quieter environment (5)
- ☐ Other (please state) (6) ____

End of Block: Block 4

Start of Block: Block 2

Goodwin L, *et al. BMJ Open* 2024; 13:e075022. doi: 10.1136/bmjopen-2023-075022

Q15 Which of the following do you feel are triggers for challenging behaviour associated with dementia (or suspected dementia) in the ED?

- ☐ Patient is in pain (1)
- ☐ Mental capacity of patient (2)
- ☐ Patient does not have their regular medication with them (3)
- ☐ Difficulty communicating with staff/being understood (4)
- ☐ Basic care needs are not met (5)
- ☐ Undiagnosed symptoms (6)
- ☐ ED waiting times (7)
- ☐ Noise of the ED environment (8)
- ☐ Not given enough time/attention by staff (9)
- ☐ Other (please state) (10) ____
- ☐ ⊗None of the above (11)

Q16 Do you feel that these instances of challenging behaviour could be **prevented**?

- ○ Yes (1)
- ○ No (2)
- ○ Sometimes (3)

Skip To: Q18 If Q16 = No

Goodwin L, *et al. BMJ Open* 2024; 13:e075022. doi: 10.1136/bmjopen-2023-075022

Q17 What do you feel could be done to **prevent** instances of challenging behaviour associated with dementia (or suspected dementia) in the ED?

- ☐ Shorter waiting times (1)
- ☐ Rapid assessment (2)
- ☐ Clearer hospital signage (3)
- ☐ Specialist input (i.e. Geriatrician) (4)
- ☐ 1:1 care from staff/carer (5)
- ☐ Written information for staff regarding patient's medical background/preferences (6)
- ☐ Quieter environment (7)
- ☐ Dimmer lights (8)
- ☐ Snacks provided (9)
- ☐ Provide distraction (i.e. music, photos, TV, puzzles, twiddle muff, other activities) (10)

Goodwin L, *et al. BMJ Open* 2024; 13:e075022. doi: 10.1136/bmjopen-2023-075022

Q18 What do you feel could be done to **de-escalate** instances of challenging behaviour associated with dementia (or suspected dementia) in the ED?

☐ Specialist input (i.e. Geriatrician) (1)

☐ 1:1 care from staff/carer (2)

☐ Written information for staff regarding patient's medical background/preferences (3)

☐ Quieter environment (4)

☐ Dimmer lights (5)

☐ Snacks provided (6)

☐ Provide distraction (i.e. music, photos, TV, puzzles, twiddle muff, other activities) (7)

☐ Pharmaceutical measures (8)

End of Block: Block 2

Start of Block: Block 5

Q19 Do you feel that there should be a separate assessment area of the ED for patients with dementia (or suspected dementia)? (please give a reason for your answer)

○ Yes (1) ____

○ No (2) ____

End of Block: Block 5

End of Survey message:

Thank you for completing this survey, your responses have been recorded.

Goodwin L, *et al. BMJ Open* 2024; 13:e075022. doi: 10.1136/bmjopen-2023-075022

If you have any questions about this project, please contact Dr Laura Goodwin (laura.goodwin@uwe.ac.uk)

Goodwin L, *et al. BMJ Open* 2024; 13:e075022. doi: 10.1136/bmjopen-2023-075022
