# Supplementary material for: How do emergency department staff respond to behaviour that challenges displayed by people living with dementia? A mixed-methods study
Source: BMJ Open. 2023 Aug 4;13(8):e075022. doi: 10.1136/bmjopen-2023-075022 (PMC10407375; doi:10.1136/bmjopen-2023-075022)
Supplement: Supplementary data [file bmjopen-2023-075022supp001.pdf]

Interview Topic Guide

Introductions, re-confirm consent. Explain aims of interview

Preliminary questions

Can you confirm your role within the emergency department (i.e. nurse, doctor, porter)?

How long have you worked in the ED?

Are there any dementia friendly initiatives used in your emergency department?

- What are these initiatives? How long have they been in place?

Have you received any staff training in relation to dementia?

- When? What did this entail? Did it include challenging behaviour? How useful was it?

Challenging behaviour

Have you experienced challenging behaviour from patients with dementia in the ED?

- YES: Can you tell us about these experiences?
  - What types of behaviours are exhibited?
  - How frequently do these instances occur?
  - Are these incidents reported?
  - Do you need other staff members to help? How many?
  - Did the patient need 1 to 1 care/support?
  - Are other patients impacted by this behaviour? In what way?
  - How are these situations de-escalated/ended?
  - How long do these situations last?
  - How long do these patients wait for a decision to be admitted or are they transferred to another area at the hospital such as a medical assessment unit?
  - Why do you think these situations occur? Any contributing factors/triggers?
  - Can you think of anything which may **prevent** these situations from occurring?
  - Can you think of anything which may **de-escalate** these situations once they are already happening?
- NO: If you have not had any experiences of this, why do you think these situations may occur? Any contributing factors/triggers?
  - Can you think of anything which may **prevent** these situations from occurring?
  - Can you think of anything which may **de-escalate** these situations once they are already happening?

Is there anything you can think of which could be changed about the ED environment in order to avoid challenging behaviour from patients with dementia?

- How can the organisation/staff/patients & their carers help?

Goodwin L, *et al. BMJ Open* 2024; 13:e075022. doi: 10.1136/bmjopen-2023-075022

Current management techniques

Have you witnessed any situations in the ED where sedation or restraint has been needed to manage challenging behaviour from patients with dementia?

Are there any assessment tools available within your ED to identify whether or not patients with dementia are likely to display challenging behaviour?

Intervention

Explain the aim to develop an intervention to prevent and/or de-escalate challenging behaviour from patients with dementia in the ED.

How do you feel about introducing this kind of intervention?

- Any possible challenges/best ways to do this?

Can you think of a way to **measure** the benefit of any new intervention?

- Physiological measures of the patient? Behavioural measures? Cognitive measures? Incident rates?
