# Supplementary material for: How do emergency department staff respond to behaviour that challenges displayed by people living with dementia? A mixed-methods study
Source: BMJ Open. 2023 Aug 4;13(8):e075022. doi: 10.1136/bmjopen-2023-075022 (PMC10407375; doi:10.1136/bmjopen-2023-075022)
Supplement: Author's manuscript [file bmjopen-2023-075022.draft_revisions.pdf]

BMJ Open is committed to open peer review. As part of this commitment we make the peer review history of every article we publish publicly available.

When an article is published we post the peer reviewers' comments and the authors' responses online. We also post the versions of the paper that were used during peer review. These are the versions that the peer review comments apply to.

The versions of the paper that follow are the versions that were submitted during the peer review process. They are not the versions of record or the final published versions. They should not be cited or distributed as the published version of this manuscript.

BMJ Open is an open access journal and the full, final, typeset and author-corrected version of record of the manuscript is available on our site with no access controls, subscription charges or pay-per-view fees (http://bmjopen.bmj.com).

If you have any questions on BMJ Open's open peer review process please email info.bmjopen@bmj.com

How do Emergency Department staff respond to behaviour that challenges displayed by people living with dementia? A mixed-methods study

Journal:	*BMJ Open*
Manuscript ID	bmjopen-2023-075022
Article Type:	Original research
Date Submitted by the Author:	24-Apr-2023
Complete List of Authors:	Goodwin, Laura; University of the West of England<br>Liddiard, Cathy; University of the West of England<br>Manning, Sera; University of the West of England<br>Benger, Jonathan; University of the West of England<br>Carlton, Edward; North Bristol NHS Trust<br>Cheston, Richard; University of the West of England<br>Hoskins, Rebecca; University Hospitals Bristol and Weston NHS Foundation Trust<br>Taylor, Hazel; University Hospitals Bristol NHS Foundation Trust, Research Design Service<br>Voss, Sarah; University of the West of England
Keywords:	Dementia, ACCIDENT & EMERGENCY MEDICINE, Hospitals, Public, Aging

SCHOLARONE™
Manuscripts

I, the Submitting Author has the right to grant and does grant on behalf of all authors of the Work (as defined in the below author licence), an exclusive licence and/or a non-exclusive licence for contributions from authors who are: i) UK Crown employees; ii) where BMJ has agreed a CC-BY licence shall apply, and/or iii) in accordance with the terms applicable for US Federal Government officers or employees acting as part of their official duties; on a worldwide, perpetual, irrevocable, royalty-free basis to BMJ Publishing Group Ltd ("BMJ") its licensees and where the relevant Journal is co-owned by BMJ to the co-owners of the Journal, to publish the Work in this journal and any other BMJ products and to exploit all rights, as set out in our licence.

The Submitting Author accepts and understands that any supply made under these terms is made by BMJ to the Submitting Author unless you are acting as an employee on behalf of your employer or a postgraduate student of an affiliated institution which is paying any applicable article publishing charge ("APC") for Open Access articles. Where the Submitting Author wishes to make the Work available on an Open Access basis (and intends to pay the relevant APC), the terms of reuse of such Open Access shall be governed by a Creative Commons licence – details of these licences and which Creative Commons licence will apply to this Work are set out in our licence referred to above.

Other than as permitted in any relevant BMJ Author's Self Archiving Policies, I confirm this Work has not been accepted for publication elsewhere, is not being considered for publication elsewhere and does not duplicate material already published. I confirm all authors consent to publication of this Work and authorise the granting of this licence.

For peer review only

How do Emergency Department staff respond to behaviour that challenges displayed by people living with dementia? A mixed-methods study

Authors:
Goodwin, L.[1], Liddiard, C.[1], Manning, S.,[1] Benger, J.[1], Carlton, E.[2], Cheston, R.[1], Hoskins, R.[3], Taylor, H.[3], & Voss, S.[1]

Institutions:
[1] University of the West of England, Bristol
[2] North Bristol NHS Trust
[3] University Hospitals Bristol and Weston NHS Foundation Trust

Corresponding author:
Laura Goodwin – laura.goodwin@uwe.ac.uk

Keywords:
Dementia, Emergency Service, Hospital, Behavioural Symptoms, Aggression

List of abbreviations:
BtC – Behaviour that challenges
ED – Emergency department

Word count:
4360

For peer review only - http://bmjopen.bmj.com/site/about/guidelines.xhtml

For peer review only

ABSTRACT

Objectives: To investigate staff experiences of, and approaches to behaviour that challenges displayed by patients with dementia in the Emergency Department.

Design: Mixed-methods study consisting of an online survey and semi-structured telephone interviews. Quantitative data were analysed and presented using descriptive statistics. Qualitative data were analysed thematically.

Setting: The Emergency Departments of three NHS Hospital Trusts in Southwest England.

Participants: Multidisciplinary NHS staff working in the Emergency Department.

Results: Fifty-two online survey responses and 13 telephone interviews were analysed. Most (24/36, 67%) survey respondents reported that they had received general training in relation to dementia in the last 2-5 years, however less than a fifth (4/23, 17%) had received any ED-specific dementia training. All (48/48) felt that behaviour that challenges could potentially be prevented, though resource constraints and practice variation were identified. Four main themes emerged from the qualitative data: (1) The "perfect storm" of the ED; (2) Behaviour that challenges is preventable with the right resources; (3) Improvisation; (4) Requirement for approaches that are specific to the Emergency Department.

Conclusions: The findings of this study suggest that Emergency Department staff do not feel that they are prepared to respond effectively to behaviour that challenges displayed by people living with dementia. Future work could adapt or develop an intervention to support Emergency Department staff in responding to behaviour that challenges.

STRENGTHS AND LIMITATIONS OF THIS STUDY

- To the best of our knowledge, this is the first study to focus specifically on staff experiences of responding to behaviour that challenges associated with dementia in United Kingdom Emergency Departments.
- A mixed-methods study design including survey and qualitative interviews provided both an overview and more detailed description of staff experiences.
- The survey had a relatively low response rate and the research was confined to one geographical area which may limited the representativeness of our findings.

FUNDING STATEMENT

The study was funded by the University Hospitals Bristol and Weston NHS Foundation Trust Above and Beyond (Charitable Trustees of UH Bristol) and UH Bristol Research Capability Funding (RCF) Grants awarded through the Research Funding Committee (2019-Spr-03).

COMPETING INTERESTS

None declared

For peer review only - http://bmjopen.bmj.com/site/about/guidelines.xhtml

BACKGROUND

In the United Kingdom (UK), there are around 944,000 people living with dementia, and this is estimated to increase to more than 1.6 million by 2050.(1) Challenging behaviour, also referred to by the Alzheimer Society and others as 'behaviour that challenges' (BtC),(2) is commonly associated with dementia and has been defined as "actions that detract from the well-being of individuals due to the physical or psychological distress they cause within the settings they are performed".(3) BtC can take many forms including aggressive physical actions (e.g., kicking, biting), shouting and verbal aggression and non-aggressive behaviour including repetitive questioning, inappropriate exposure and resistance to care. This behaviour often stems from an unmet need – for instance to reduce distress or frustration or to maintain a sense of wellbeing. Both the person living with dementia and the people around them are likely to be impacted by their behaviour.(4)

An increase in the number of people living with dementia is reflected in emergency hospital admissions. In England there was a rise of 35% in emergency admissions for people with dementia during 2017-18, compared with 2012-13.(5) More than half of the people who had a diagnosis of dementia in 2017-18 were admitted to hospital as an emergency at least once in that year.(5)

In a busy Emergency Department (ED), the physical and medical aspects of care such as completing observations and administering medication may become prioritised over less technical elements (for example helping with toileting or food/drink),(6) and this may result in unmet needs for older people living with dementia.(7) For instance, the loud and often crowded environment of the ED can result in sensory overload for people living with dementia,(8,9) and may lead to dehydration, ineffective pain management exacerbating other unmet needs with an increased risk of BtC.(10–12)

Behaviour that challenges can be difficult to respond to for ED staff, who need to ensure safety and prompt treatment for all patients. In a retrospective audit by Tropea et al. (2017),(13) 39% of people living with dementia attending an ED in Australia exhibited behaviour that challenges, with aggression being the most common (35%). Despite the importance of dementia training being recognised by Royal College of Emergency Medicine,(14) there are no standardised processes for managing patients living with dementia or cognitive impairment in UK EDs. Without training and resources to prevent or respond to challenging behaviour associated with dementia, ED staff have previously relied on sedating or restraining patients, which is often distressing for patients, family members and staff and likely to lead to adverse outcomes.(13,15)

Various approaches are available to hospital staff for responding to challenging behaviour of people living with dementia: creating a calm, quiet physical environment;(16) sensory stimulation such as light and music therapy;(17) distraction activities such as video, music boxes or dolls;(18) accessing 'hospital ready' information about the patient and their wishes.(19) However, there is little research on the effectiveness of prevention or de-escalation interventions in EDs,(15) where there may be unique challenges to implementing some of these approaches. Therefore, this study aimed to investigate UK National Health Service (NHS) staff experiences of responding to BtC, associated with dementia, in the ED. The aims of this project were:

1. To explore staff experiences of, and approaches to behaviour that challenges associated with dementia in the ED environment.

For peer review only - http://bmjopen.bmj.com/site/about/guidelines.xhtml

2. To explore ED staff opinions of using de-escalation techniques within an ED setting and any perceived barriers and facilitators to introducing new interventions.

METHODS

Study design

Mixed-methods study consisting of an online survey and semi-structured interviews. Both the survey and interviews were open to all staff working in three NHS Hospital Trust EDs in Southwest England. Convenience sampling was used: an email promoting the study (and including the survey link) was sent to all ED staff by local collaborators at each Trust and disseminated through bulletins and posters with a quick response (QR) code for the survey displayed in ED staff rooms at participating sites. Recruitment adverts included an offer of a £10 voucher for those who completed an interview.

The survey was hosted on the 'Qualtrics' platform (Provo, Utah) and accessed through the disseminated links and QR codes. Participants were required to confirm consent electronically before the first survey question was presented. As the survey was exploratory, a sample size calculation was not required.

Eligible participants were invited to contact the lead author to take part in an interview. Potential interview participants received a participant information sheet, privacy notice and consent form via email. Verbal confirmation of consent was audio-recorded at the start of the telephone interview. We aimed to recruit 10-15 participants, guided by information power with a narrow aim and specific study population.(20)

Data collection

Survey

The open online survey (Supplemental file 1) was developed by the study team and reviewed by members of our Patient and Public Involvement (PPI) group. It was then piloted for usability and technical functionality by members of the study team. The survey was open between November 2020 and November 2021. A participant information sheet, privacy notice and consent form were integrated into the first page of the survey. The survey contained questions about the extent and nature of the issues associated with caring for patients with dementia who may exhibit behaviour that challenges in an ED setting. Questions with open free-text responses were included alongside questions with Likert scales and multiple-choice responses.

Interviews

The participant information sheet, consent form and topic guide (Supplemental file 2) were developed in collaboration with the study team and our PPI group. Semi-structured interviews were used to investigate staff experiences of responding to BtC associated with dementia in the ED and any prevention/de-escalation techniques used. A single interview was held with each participant, which was conducted via telephone at a time convenient for the participant. Interviews were held between October 2020 and November 2021 by experienced qualitative researchers (LG and CL) and audio-recorded via Skype for Business. Interviews lasted an average of 45 minutes. Audio files were transcribed verbatim.

Analysis

Survey data were exported from Qualtrics to a Microsoft Excel spreadsheet. Quantitative data were analysed and presented using descriptive statistics. Free-text data were summarised and categorised.

For peer review only - http://bmjopen.bmj.com/site/about/guidelines.xhtml

Interview transcripts were coded and analysed by CL and LG, supported by NVivo 12 software. Data were analysed thematically through a well-established iterative process of data reduction, constant comparison, organisation and understanding.(21) The researchers read the transcripts several times and then coded selections of text to represent instances of a concept. Codes were then reviewed in terms of their relationship to other codes and combined to create more developed themes. From this analysis, distinctions could be made between the different levels of themes.

Patient and public involvement

PPI involvement was gained from a local support group for people living with dementia and their carers in Bristol, who have experience of attending local EDs as/with a person living with dementia. The group provided feedback on the study aims, design and recruitment, and supported the research team in designing all patient-facing documents.

RESULTS

Participant characteristics

A total of 52 responses to the survey were received from ED staff (Table 1). Thirteen interviews were conducted (Table 2).

Table 1. Survey respondent characteristics (n=52)

**Characteristic**		**No. (%) of respondents**
Role		
	Doctor	22 (42%)
	Nurse	19 (37%)
	Admin staff	1 (2%)
	Porter	1 (2%)
	Other	9 (17%)
Approximate experience working in ED (years)		
	<1	6 (12%)
	1-2	13 (25%)
	3-5	8 (15%)
	6-10	9 (17%)
	11-15	8 (15%)
	16-20	5 (10%)
	21+	3 (6%)

Table 2: Interview participant characteristics (n=13)

**Characteristic**		**No. (%) of respondents**
Role		
	Doctor	4 (31%)
	Nurse	8 (62%)
	Other	1 (7%)
Gender		
	Female	10 (77%)
	Male	3 (23%)
Approximate experience working in ED (years)		
	≤3	4 (31%)

For peer review only - http://bmjopen.bmj.com/site/about/guidelines.xhtml

	4-6	5 (39%)
	7-9	2 (15%)
	10+	2 (15%)

Four main themes were identified from the qualitative data: (1) The "perfect storm" of the ED; (2) Behaviour that challenges is preventable with the right resources; (3) Improvisation; (4) Requirement for approaches that are specific to the Emergency Department. These are reported in narrative form, supported by quotes, and woven into the quantitative results below.

Theme 1: The "perfect storm" of the ED

The significant challenges that the ED setting presents to people with dementia was described by all interview participants. Survey data indicated that almost all (49/52, 94%) respondents had experienced BtC in the ED from patients living with dementia. Interviewees perceived the 'perfect storm' of the ED to be made up of triggers (physiological and environmental), the ED's clinical approach to patients, and a lack of resources.

The patient being in pain was most commonly ranked as the number one trigger for BtC by survey participants (14/46, 30%). During interviews however, most participants described the hospital environment itself as the dominant triggering factor for BtC due to an absence of familiar faces and routine. Most participants reported that there to were factors unique to the ED (in comparison to other hospital settings) which compounded patients' unmet needs such as: constant bright lighting; the busy nature of the department; long waits; open areas that pose a risk of absconding; staff having less time to get to know their patient.

> It's noisy, it's bustling, it's confusing, you [the patient] often feel isolated, there are people walking past you all the time looking at you. There are strangers coming in and out. I mean everything in ED plays against dementia. You know, for those of us that are used to the chaos of the place it can still be challenging on a bad day, but to somebody with dementia it must be a nightmare. It's got everything going against it. (P011)

Inadequate information transfer was also highlighted as a resource issue for effectively managing BtC in the ED. Interview participants reported incomplete handovers from ambulance staff, absent personalised care plans, and less chance of family/friends being able to communicate the patient's needs, due to restrictions on visitors since related to the Covid-19 pandemic.

Some participants described how the primary function of the ED is to focus on the patient's presenting clinical condition (for example stemming the bleeding from a fall, or providing treatment for blood pressure), which meant that they were often unable to remove the environmental triggers in the ED for patients living with dementia, as they were necessary for providing safe medical care.

> Everybody needs to back off and leave them for a while because I think any form of intervention only escalates the situation. The trouble is, of course, we are under time pressures and our focus is on getting the injury sorted. (P011)

For peer review only - http://bmjopen.bmj.com/site/about/guidelines.xhtml

Within this ‘perfect storm’, interviewees recalled numerous experiences of BtC in the ED, with many reporting instances occurring on every shift. Survey data reflected the interviews, where the majority of participants (36/46, 78%) reported that BtC occurred on a weekly basis in their ED. The most common types of behaviour reported by participants included ‘confusion’ (45/46; 98%), ‘wandering’ (43/46; 94%), ‘agitation’, (43/46; 94%) and ‘verbal aggression’ (42/46; 91%). Interview participants described instances of all of these behaviours within their ED, and reported concerns regarding patient safety and staff resource.

> The most frequent experience that happens with a patient with dementia is that they come into the emergency department and they wander, and unfortunately we don’t have a dedicated member of staff to stay with them. So, it’s scary because as a nurse you feel that they are in danger. (P007)

Some behaviours were reported as being more challenging in an ED environment, and participants expressed anxiety around gaps in dementia care in the ED.

> I don’t think there’s any areas that are 100% safe. I know you can’t be with dementia but it’s quite risky in ED. But then they’re only supposed to be there for four hours and then go onto a ward. But that obviously doesn’t always happen with, you know, lack of beds. (P006)

Interviewed staff reported that prevention and de-escalation of BtC could be difficult to maintain in the ED environment and that, if responded to ineffectively then behaviours found to be challenging by staff could continue throughout the patient’s entire ED stay.

> Nine times out of ten [the BtC lasts] until they get to a ward, or until they are picked up by transport. (P001)

> I would say there are nights where we don’t really get people settled at any point. (P012)

Some interviewees described how the challenge of managing BtC worsened at night, as people living with dementia often experienced sleep deprivation and were unable to settle in the ED environment. One reported the rate of falls to be higher at night, and noted difficulties escalating patients or getting support from dementia teams during this time.

Theme 2: Behaviour that challenges is preventable with the right resources

Survey respondents indicated they felt that instances of BtC could either be prevented (13/48 30%) or sometimes prevented (35/48, 73%) in the ED. One-to-one care from staff or a carer was ranked as the intervention most likely to prevent BtC (18/44, 41%), followed by rapid assessment of the patient (10/44, 23%). For de-escalating BtC, one-to-one care was also the highest-ranking technique (25/44, 57%), followed by the provision of a distraction (7/44, 16%) or a quieter environment (7/44, 16%). However, participants in both the survey and interviews highlighted the lack of resources available to provide these types of interventions, noting concerns regarding crowded EDs, understaffing and long ambulance wait times.

> It’s the endless resources isn’t it, that we don’t have? If there were a team of Dementia Nurses in the hospital that you know, Healthcare Assistants even, that could just come and sit with the patients, engage

For peer review only - http://bmjopen.bmj.com/site/about/guidelines.xhtml

them, in looking at that magazine, listening to that music, you know, or whatever it is, if it's just sitting and chatting with them. (P009)

Despite a common acknowledgment of the unique challenges of the ED for people living with dementia, less than a fifth (4/23, 17%) of survey respondents had received dementia training that was specific to the ED context. None of the interviewees reported having had any ED-specific dementia training.

While most survey respondents (24/36, 67%) reported that they had received general training in relation to dementia in the last 2-5 years, a large proportion (11/23, 48%) found it only 'moderately useful'. This was reflected in interview data, where general dementia training was given mixed reviews in terms of its usefulness.

Most interviewees could recall generic dementia-friendly initiatives that had been implemented in their ED. One of the initiatives, music, was enthusiastically highlighted as being particularly helpful to patients, although frustration was expressed at how quickly equipment would go missing. Other de-escalation techniques were also perceived as useful to staff and patients, but interview participants noted that such techniques often needed an investment of time and staff to facilitate them, which was not always possible. Indeed, limitations (most often linked to resource issues) were described for most of the initiatives described positively by staff (Table 3).

Table 3. Existing dementia-friendly initiatives recalled by interview participants.

Initiative	Limitation(s)
Reminders of the past: designing corridors with pictures of past-times and/or old films played on a TV	• They become out of date.
Colouring Books	• Not always in stock.
Box of distractions	• Requires space/tables to use.<br>• Can require a staff member to facilitate their use.<br>• Not available during Covid-19 as has to go through infection control.
Providing food and drink	• Short-term solution<br>• Hot food not always available in ED.<br>• White plates can cause confusion with some (white) foods.
'This is Me' (22) or similar: document containing information about the patient's needs and wishes	• Patient does not always come in with it.<br>• Specific paperwork required to create one is not in stock<br>• No time to create one in ED setting.<br>• Suggestions within it may not be appropriate for the ED setting.

For peer review only - http://bmjopen.bmj.com/site/about/guidelines.xhtml

Music	• Equipment went missing.
Family member/carer	• Covid-19 restrictions reduced this.
Individual cubicles	• Not always safe, as unable to see the patient.<br>• Many cubicles not available as used for patients with Covid-19 or other infectious conditions

The majority of survey respondents (30/45, 67%), felt that there should be a separate assessment area of the ED for patients with dementia. Free-text comments added that the ideal space should be quiet, where the lights can be dimmed and attended by staff trained in dementia. From respondents that did not think there should be a separate assessment area (33% 15/45), free-text comments showed support for this type of area in an "ideal world" but reported that the logistical and resource challenges this would present to enable patients to be safe there was likely to render the initiative unfeasible.

Theme 3: Improvisation

While most survey respondents (23/36, 64%) reported that they covered information about how to manage BtC in their general dementia training, there was variation in the management of BtC by the ED staff surveyed. The most used approaches to managing BtC were distraction techniques (37/46, 81% reported using them 'always' or 'most of the time'), and the use of family and/or carers (20/46, 43% reported using them 'always' or 'most of the time').

Some interviewees reported using their own individual approaches to preventing and de-escalating BtC, learnt from other professional roles or their personal life. One described 'messing-up' sheets and asking patients to fold them, or offering bandages to squeeze whilst carrying out a procedure to keep their hands occupied.

> Everyone uses their own sort of techniques. No-one's really said you can or can't do this, but you just have to improvise with what you've got and it's difficult when you haven't got a lot to help you. (P011)

Interview participants reported that an inconsistent approach to managing BtC often resulted in ineffective or non-sustained management of behaviour within the ED, and that this became particularly problematic when it led to patients resisting medical care. Indeed, the majority of survey respondents (87%, 40/46) reported that they had experienced physical resistance to care when supporting people living with dementia in the ED. Interview participants reported that this often meant that procedures were either minimised or postponed, or described some kind of 'battle' with the patient to complete the procedure; causing distress for both patient and staff.

> I had a lady yesterday, who…needed a Lateral Flow test. So, you know, to go and stick a thing up her nose, she didn't want that done. And it was a horrible thing to have to do and, in the end, I gave up with it, because it was just distressing her too much... But yeah, she was grabbing and pinching [me] and you know, she, she was quite up for giving you a hearty thump at the end of it. (P009)

For peer review only - http://bmjopen.bmj.com/site/about/guidelines.xhtml

When asked directly about the use of physical restraint to manage BtC in the ED, most survey respondents (36/46, 78%) reported that they never used it. However, there seemed to be some differences in how the word 'restraint' was interpreted by interview participants, which often led to contradictory accounts during interviews. While most interviewees said that restraint was only very rarely used, when questioned directly, many described using or witnessing some form of restraint frequently (to administer care) when talking more generally about their experiences of managing BtC in the ED.

> I've not seen anyone have to be physically restrained or anything like that. I think we try to kind of implement things in place before anything like that would need to happen. (P002)

> For simple things like taking a blood test…you'll often hold the patient's hand down, or arm down, and they can't consent. (P005)

There was also a contradiction between survey and interview responses when reporting the use of pharmaceutical measures to manage BtC in the ED. Interviewees were unified in their perception that the use of pharmaceutical measures to manage BtC in the ED was very rare, whilst the majority (38/46, 83%) of survey respondents reported that this approach was 'sometimes' used. Again, there seemed to be some differences in the interpretation of the wording, which meant that mild sedation to complete a medical procedure was not always viewed as a pharmaceutical measure to manage BtC.

Some interview participants described an accelerated approach to completing medical assessments/procedures to minimise the chance of the patient showing physical resistance to care, even if this meant completing tasks without explicit consent.

> It's horrendous, but you just do things. You almost don't ask, if you're doing these things… I might say, 'oh I'm going to exam your eyes, that's okay?' But you just roll off the natural things, and the nurses do them, and you're doing it out of best interest, but sometimes it's easier to be like 'oh, they don't necessarily understand' and you just crack on and do it... You're just trying to be, like, she probably won't understand the blood test and things, but I think she really needs it, she might have a raging infection, we need to do this. (P005)

Theme 4: Requirement for approaches that are specific to the Emergency Department
There was overwhelming support from interview participants regarding the potential introduction of an ED-specific intervention to support staff in managing BtC. In fact, one participant had developed their own dementia training package, specific to the ED. This training aimed to encourage staff to consider the potential trigger(s) of the BtC (i.e., toileting needs, discomfort/pain) and ways to minimise these triggers, whilst the patient was in the ED.

A number of participants put forward the idea of a simple check-list or escalation toolkit for care, which could use a *'step-wise approach that will guide you in both recognising and dealing with these behaviours'* (P004). Participants said that a formal tool would increase the consistency of approach to BtC across the ED and provide something to follow when behaviour was escalating. One interviewee described their vision of what an 'escalation tool' could look like:

> If it was like a scale of one to ten, the first thing is make sure they're not in pain and then the second thing is do they need the toilet, almost like a tick

For peer review only - http://bmjopen.bmj.com/site/about/guidelines.xhtml

> box of things you need to consider, and then you work your way up…from basic things to, kind of, more difficult things to implement. Then you could tick them off almost - what you've done and at what point it de-escalated. So then it would potentially help with their care in the future when they're on the wards or if they came back into ED. (P002)

Another suggested that a new intervention should give more emphasis to preventing triggers for BtC, rather than focusing solely on de-escalation.

> I guess the bit we don't talk about is preventing those behaviours happening in the first place, and how to make our department a place that prevents things escalating…We wait for it to escalate and then we think about, is this patient comfortable? Are they in a quiet enough environment? Do they have food and drink? (P012)

Other suggestions included better identification of patients who may be living with dementia, specialist ED-specific training for staff, and improved co-ordination with other services and organisations. For example, participants reported that there could be better handover from care-homes and ambulance services, including multi-service utilisation of an electronic version of the 'This is Me'(22) document, or arranging for home-comforts to be brought to the ED with the patient.

> We don't think enough about them having something from their home or from their care home, like a blanket or something that they find familiar, that has the right smell. (P007)

There were some potential barriers put forward by ED staff when implementing a new intervention. Interview participants advised that any new initiative should not need too much space or time, be complex, or *'another piece of paperwork to fill out'* (P012). They also warned that measuring the impact of any new intervention may be challenging.

Facilitators to a new intervention, put forward by interview participants, included buy-in from key stakeholders and emphasis on how the intervention would help patients. One interviewee suggested the use of face-to-face study days to explain the need for a new tool, whilst another emphasised the need for any information to be distributed to all ED staff who would come into contact with patients (i.e. including porters, receptionists, security guards and health care assistants).

DISCUSSION

To the best of our knowledge, this is the first study to specifically focus on staff experiences of managing BtC in the ED environment in the UK. A mixed-methods study design combining survey and qualitative interviews provided both a wide overview as well as a rich description of staff experiences. Important weaknesses of our study include the relatively low response rate and the fact that the research was confined to one geographical area which will have limited the representativeness of our findings. We used a convenience sample of volunteers, and so the experiences and views on which our research is founded may not be representative of all ED staff.

We found several challenges associated with managing BtC associated with dementia in the ED. These included the concept of the ED as a 'perfect storm' for behavioural triggers, a lack of resources to effectively prevent and de-escalate BtC within the ED, and inconsistent approaches

For peer review only - http://bmjopen.bmj.com/site/about/guidelines.xhtml

to managing BtC, leading to escalation of behaviour. Staff were positive about the idea of introducing ED-specific interventions to prevent/de-escalate BtC and offered a number of suggestions for what this might include.

The description of the ED as a 'perfect storm' is well supported in the literature regarding dementia.(23) Our findings suggest a lack of ED-specific training and resources. While a number of different interventions have been found to be helpful in the general hospital setting, the effectiveness of these approaches in the ED is unknown due to a lack of research.(15) Participants in our study noted that a lack of ED-specific training and resources led to variation in the techniques used by staff to respond to and de-escalate BtC, which in turn led to ineffective management; staff were left to improvise with the resources they had, often attempting to intervene once behaviours had already escalated substantially.

In line with previous literature, our findings suggest that ineffective management of BtC sometimes resulted in physical and pharmacological restraint being used by staff in the ED.(13) However, there seemed to be differences in the interpretation of words such as 'restraint' and 'sedation' by participants; some staff did not perceive physical measures used to facilitate minor procedures as formal restraint. Similar findings were noted in a qualitative study of Australian emergency nurses who did not perceive manual restraint for minor injuries care as "formal" restraint.(24)

Our findings reflect the high frequency of BtC in the ED reported in other literature.(13) While staff felt that that some incidents of BtC could be prevented, this was mainly through resource-intense approaches (i.e. 1:1 care or a dedicated space for triage and assessment), which participants felt was unlikely to prove realistic in the context of resource limitations. The under-use of family and friends was noted in research prior to Covid-19,(25) and is likely to have been exacerbated during and after the pandemic.

Participants in our study expressed support for a specific checklist or flowchart approach to guide care and behaviour management in the ED. These have been used successfully in a variety of healthcare settings to act as an aid to decision-making or memory recall, including responding to BtC for people living with dementia in residential care.(26,27)

CONCLUSION

The findings of this study suggest that Emergency Department staff do not feel they are prepared to effectively manage BtC in the ED due to the unique combination of triggers presented by the environment, and the lack of resources available in this setting. This is an increasing issue, due to the rising numbers of ED admissions for people living with dementia. Future work could adapt or develop an intervention to effectively support ED staff in managing BTC associated with dementia.

For peer review only - http://bmjopen.bmj.com/site/about/guidelines.xhtml

STATEMENTS

Acknowledgments

We would like to thank the ED staff who took part in interviews for this research, as well as those who promoted this study.

Author Contributions

SV, SM, EC, RC, RH, HT and JB participated in study conception, design and delivery. LG and CL participated in study coordination, performed and coded the interviews, analysed the data and drafted the manuscript. All authors were responsible for the critical revision of the manuscript for publication and approved the final version. SV acts as guarantor.

Patient and Public Involvement

Patients and/or the public were involved in the design, or conduct, or reporting, or dissemination plans of this research. Refer to the Methods section for further details.

Patient consent for publication

Not required

Ethics approval

This study involves human participants and ethical approval was granted by the NHS Health Research Authority (20/HRA/0587) as well as the University of the West of England (UWE Bristol) Faculty of Health and Applied Sciences Research Ethics Committee (HAS-NAM-18-027). Participants gave informed consent to participate in the study before taking part.

Provenance and peer review

Not commissioned; externally peer reviewed.

Data availability statement

Data are available upon reasonable request.

For peer review only

REFERENCES

1. Alzheimer's Research UK. Dementia Statistics Hub: Prevalence projections in the UK [online]. 2022. https://dementiastatistics.org/statistics/prevalence-projections-in-the-uk-2/ (accessed 17 March 2023).
2. Alzheimer's Society. How does dementia change a person's behaviour? [online]. 2021. https://www.alzheimers.org.uk/about-dementia/symptoms-and-diagnosis/symptoms/behaviour-changes#content-start (accessed 17 March 2023).
3. James IA, Jackman L. Understanding behaviour in dementia that challenges: A guide to assessment and treatment. London: Jessica Kingsley Publishers 2017.
4. Cohen-Mansfield J. Agitated behaviors in the elderly: II. Preliminary results in the cognitively deteriorated. J Am Geriatr Soc 1986;34(10):722–7.

For peer review only - http://bmjopen.bmj.com/site/about/guidelines.xhtml

5. Alzheimer's Society. Analysis of NHS England's Hospital Episode Statistics dataset 2012/13 to 2017/18. [online]. 2020. www.alzheimers.org.uk/sites/default/files/2020-01/Hospital%20Admissions%202012-18%20for%20people%20with%20dementia%20Alzheimer%27s%20Society%202020.pdf (accessed 17 March 2023).

6. Mollaoğlu M, Çelik P. Evaluation of emergency department nursing services and patient satisfaction of services. J Clin Nurs 2016;25(19–20):2778–85.

7. Parke B, Hunter KF, Marck PB. A novel visual method for studying complex health transitions for older people living with dementia. Int J Qual Methods 2015;14(4):1609406915614150.

8. Silwanowicz RM, Maust DT, Seyfried LS, et al. Management of older adults with dementia who present to emergency services with neuropsychiatric symptoms. Int J Geriatr Psychiatry 2017;32(12):1233–40.

9. Clevenger CK, Chu TA, Yang Z, et al. Clinical care of persons with dementia in the emergency department: a review of the literature and agenda for research. J Am Geriatr Soc 2012;60(9):1742–8.

10. Burgstaller M, Mayer H, Schiess C, et al. Experiences and needs of relatives of people with dementia in acute hospitals—A meta-synthesis of qualitative studies. J Clin Nurs 2018;27(3–4):502–15.

11. Digby R, Lee S, Williams A. The experience of people with dementia and nurses in hospital: an integrative review. J Clin Nurs 2017;26(9–10):1152–71.

12. Digby R, Williams A, Lee S. Nurse empathy and the care of people with dementia. Australian Journal of Advanced Nursing 2016;34(1):52–9.

13. Tropea J, LoGiudice D, Liew D, et al. Caring for people with dementia in hospital: findings from a survey to identify barriers and facilitators to implementing best practice dementia care. Int Psychogeriatr 2017;29(3):467–74.

14. Royal College of Emergency Medicine. Best Practice Guidelines, Emergency Department Care [online]. 2017. http://www.rcem.ac.uk/docs/College%20Guidelines/Emergency%20Department%20Care%20-%20BP%20Guidance%20-%20Jul%202017%20-%20v6.pdf (accessed 17 March 2023).

15. Kennedy M, Koehl J, Shenvi CL, et al. The agitated older adult in the emergency department: a narrative review of common causes and management strategies. J Am Coll Emerg Physicians Open 2020;1(5):812–23.

16. Bracken-Scally M, Keogh B, Daly L, et al. Assessing the impact of dementia inclusive environmental adjustment in the emergency department. Dementia 2021;20(1):28–46.

17. Ricker JR, Mulligan M. Activity kits as a first line intervention to care for individuals with dementia. Geriatr Nurs (Minneap) 2017;38(6):604–5.

18. Fisher JE, Buchanan JA. Presentation of preferred stimuli as an intervention for aggression in a person with dementia. Behavior Analysis: Research and Practice 2018;18(1):33.

For peer review only - http://bmjopen.bmj.com/site/about/guidelines.xhtml

19. Parke B, Hunter KF. The dementia-friendly emergency department: An innovation to reducing incompatibilities at the local level. Healthcare Management Forum. Los Angeles, CA: SAGE Publications 2017: 26–31.

20. Malterud K, Siersma VD, Guassora AD. Sample size in qualitative interview studies: guided by information power. Qual Health Res 2016;26(13):1753–60.

21. Braun V, Clarke V. Using thematic analysis in psychology. Qual Res Psychol 2006;3(2):77–101.

22. Alzheimer's Society. This is me online]. 2010. https://www.alzheimers.org.uk/sites/default/files/2020-03/this_is_me_1553.pdf (accessed 20 March 2023).

23. Parke B, Hunter KF, Schulz ME, et al. Know me–A new person-centered approach for dementia-friendly emergency department care. Dementia 2019;18(2):432–47.

24. Chapman R, Ogle KR, Martin C, et al. Australian nurses' perceptions of the use of manual restraint in the Emergency Department: a qualitative perspective. J Clin Nurs 2016;25(9–10):1273–81.

25. Woods DL, Buckwalter K. Taking another look: Thoughts on behavioral symptoms in dementia and their measurement. Healthcare 2018;6(4):126.

26. Kramer HS, Drews FA. Checking the lists: A systematic review of electronic checklist use in health care. J Biomed Inform 2017;71:6–12.

27. Alzheimer's Society. Optimising treatment and care for people with behavioural and psychological symptoms of dementia: A Best Practice Guide for Health and Social Care Professionals [online]. 2011. https://www.alzheimers.org.uk/sites/default/files/2018-08/Optimising%20treatment%20and%20care%20-%20best%20practice%20guide.pdf?downloadID=609 (accessed 20 March 2023).

For peer review only

For peer review only - http://bmjopen.bmj.com/site/about/guidelines.xhtml

Dementia in the ED Survey Questions

Start of Block: Default Question Block

Welcome to 'Managing challenging behaviour related to patients in Dementia in the Emergency Department (ED)' online survey. This survey should take no longer than 5 minutes to complete. Before taking part, please read the information and consent form below, and click on the "I consent" button at the bottom of the page if you understand the information and freely consent to participate in the study.

INFORMATION AND CONSENT FORM

This survey is designed to understand ED staff experiences of challenging behaviour from patients with dementia and what interventions they have found useful. This is being led by Dr Sarah Voss and Dr Laura Goodwin of the University of the West of England, Bristol. It has been approved by the University of the West of England Research Ethics Committee.

The aim of this survey is to discover how staff in an ED department manage challenging behaviour exhibited by people with dementia in order to inform the development of a standardised de-escalation tool. These patients can often feel confused and disorientated in a hospital setting and may not be able to express pain or basic care needs. It is important they receive quality care and are as comfortable as possible. Therefore, we are interested to know why these patients may become agitated and how they are managed effectively by staff to avoid distress. The findings from this survey will be combined with data from interviews with ED staff, and may lead on to the development of an intervention to manage challenging behavior from patients with dementia in the ED.

You have been invited to take part because you are a member of staff working in the Emergency Department at either University Hospitals Bristol NHS Foundation Trust, North Bristol NHS Trust, or Royal United Hospitals Bath NHS Foundation Trust.

Participation will involve answering a series of questions about your experiences of challenging behaviour associated with dementia, whilst working in the ED. Survey participation is entirely voluntary, and there are no consequences of either choosing or not choosing to participate. You can stop being part of the study at any time, without giving a reason, but we will keep information about you that we already have. Your rights to access, change or move your information are limited, as we need to manage your information in specific ways in order for the research to be reliable and accurate. If you withdraw from the study, we will keep the information that we have already obtained. You can find out more about how we use your information by contacting dataprotection@uwe.ac.uk or by following this link:

For peer review only - http://bmjopen.bmj.com/site/about/guidelines.xhtml

www.hra.nhs.uk/information-about-patients/

Please read the following Privacy Notice before continuing.

For more information and advice, you may contact Dr Sarah Voss by phone (0117 328 8906) or email (sarah.voss@uwe.ac.uk). Alternatively you can contact Dr Laura Goodwin (laura.goodwin@uwe.ac.uk).

Q2 Do you consent to taking part in this study?

◯ I consent (1)

◯ I do not consent (2)

End of Block: Default Question Block

Start of Block: Block 3

Q3 Please select which job title best describes your role:

◯ Nurse (1)

◯ Doctor (2)

◯ Porter (3)

◯ Security staff (4)

◯ Receptionist (5)

◯ Admin staff (6)

◯ Other (please state) (7) ____

For peer review only

For peer review only - http://bmjopen.bmj.com/site/about/guidelines.xhtml

Q4 How long have you worked in an Emergency Department?

- Less than a year (1)
- 1-2 years (2)
- 3-5 years (3)
- 6-10 years (4)
- 11-15 years (5)
- 16-20 years (6)
- 21-30 years (7)
- 30 years+ (8)

End of Block: Block 3

Start of Block: Block 1

Q5 Have you ever received staff training in relation to dementia?

- Yes (1)
- No (2)

Skip To: End of Block If Q5 = No

For peer review only

For peer review only - http://bmjopen.bmj.com/site/about/guidelines.xhtml

Q6 When did you receive this training? *(if you have received more than one lot of training, please indicate when you received the **most recent** training)*

◯ In the last year (1)

◯ In the last 2-5 years (2)

◯ 6-10 years ago (3)

◯ 10+ years ago (4)

Q7 Did this training involve information about how to manage challenging behaviour associated with dementia?

◯ Yes (1)

◯ No (2)

Skip To: End of Block If Q7 = No

Q8 How useful was this information?

◯ Extremely useful (1)

◯ Very useful (2)

◯ Moderately useful (3)

◯ Slightly useful (4)

◯ Not at all useful (5)

End of Block: Block 1

Start of Block: Block 4

For peer review only - http://bmjopen.bmj.com/site/about/guidelines.xhtml
For peer review only

UWE Bristol University of the West of England

Q9 Have you experienced challenging behaviour from patients with dementia (or suspected dementia) in the ED?

- Yes (1)
- No (2)

Skip To: End of Block If Q9 = No

Q10 How frequently would you say that you experience/have to manage challenging behaviour associated with dementia (or suspected dementia) in the ED?

- Daily (1)
- Weekly (2)
- Monthly (3)
- 2-3 times a year (4)
- Less than 3 times a year (5)

Q11 How many staff members are usually required to manage instances of challenging behaviour associated with dementia (or suspected dementia) in the ED?

- 1 (1)
- 2 (2)
- 3 (3)
- more than 3 (4)

For peer review only - http://bmjopen.bmj.com/site/about/guidelines.xhtml

Q12 Please tick all words which describe your experiences of challenging behaviour associated with dementia (or suspected dementia) in the ED.

- ▢ Verbal aggression (1)
- ▢ Physical assault (2)
- ▢ Agitation (3)
- ▢ Frustration (4)
- ▢ Communication difficulties (5)
- ▢ Use of swear words (6)
- ▢ Physical resistance to care (7)
- ▢ Emotional distress (8)
- ▢ Confusion (9)
- ▢ Wandering (10)
- ▢ Disorientation (11)
- ▢ Other (please state) (12) ____

For peer review only

For peer review only - http://bmjopen.bmj.com/site/about/guidelines.xhtml

Q13 How is this behaviour usually managed?

- ☐ Pharmaceutical measures (1)
- ☐ Distraction techniques (2)
- ☐ Use of family members/carers (3)
- ☐ Restraint (4)
- ☐ Moving the patient to a quieter environment (5)
- ☐ Other (please state) (6) ____

Q14 In the last three months, which of the following behaviour management practices have you observed?

- ☐ Pharmaceutical measures (1)
- ☐ Distraction techniques (2)
- ☐ Use of family members/carers (3)
- ☐ Restraint (4)
- ☐ Moving the patient to a quieter environment (5)
- ☐ Other (please state) (6) ____

End of Block: Block 4

Start of Block: Block 2

For peer review only - http://bmjopen.bmj.com/site/about/guidelines.xhtml

Q15 Which of the following do you feel are triggers for challenging behaviour associated with dementia (or suspected dementia) in the ED?

- ☐ Patient is in pain (1)
- ☐ Mental capacity of patient (2)
- ☐ Patient does not have their regular medication with them (3)
- ☐ Difficulty communicating with staff/being understood (4)
- ☐ Basic care needs are not met (5)
- ☐ Undiagnosed symptoms (6)
- ☐ ED waiting times (7)
- ☐ Noise of the ED environment (8)
- ☐ Not given enough time/attention by staff (9)
- ☐ Other (please state) (10) ____
- ☐ ⊗ None of the above (11)

Q16 Do you feel that these instances of challenging behaviour could be **prevented**?

- ○ Yes (1)
- ○ No (2)
- ○ Sometimes (3)

Skip To: Q18 If Q16 = No

For peer review only - http://bmjopen.bmj.com/site/about/guidelines.xhtml

UWE Bristol University of the West of England

Q17 What do you feel could be done to **prevent** instances of challenging behaviour associated with dementia (or suspected dementia) in the ED?

- Shorter waiting times (1)
- Rapid assessment (2)
- Clearer hospital signage (3)
- Specialist input (i.e. Geriatrician) (4)
- 1:1 care from staff/carer (5)
- Written information for staff regarding patient's medical background/preferences (6)
- Quieter environment (7)
- Dimmer lights (8)
- Snacks provided (9)
- Provide distraction (i.e. music, photos, TV, puzzles, twiddle muff, other activities) (10)

For peer review only

For peer review only - http://bmjopen.bmj.com/site/about/guidelines.xhtml

Q18 What do you feel could be done to **de-escalate** instances of challenging behaviour associated with dementia (or suspected dementia) in the ED?

- ☐ Specialist input (i.e. Geriatrician) (1)
- ☐ 1:1 care from staff/carer (2)
- ☐ Written information for staff regarding patient's medical background/preferences (3)
- ☐ Quieter environment (4)
- ☐ Dimmer lights (5)
- ☐ Snacks provided (6)
- ☐ Provide distraction (i.e. music, photos, TV, puzzles, twiddle muff, other activities) (7)
- ☐ Pharmaceutical measures (8)

End of Block: Block 2

Start of Block: Block 5

Q19 Do you feel that there should be a separate assessment area of the ED for patients with dementia (or suspected dementia)? (please give a reason for your answer)

- ○ Yes (1) ____
- ○ No (2) ____

End of Block: Block 5

End of Survey message:

Thank you for completing this survey, your responses have been recorded.

If you have any questions about this project, please contact Dr Laura Goodwin (laura.goodwin@uwe.ac.uk)

For peer review only - http://bmjopen.bmj.com/site/about/guidelines.xhtml

Interview Topic Guide

Introductions, re-confirm consent. Explain aims of interview

Preliminary questions

Can you confirm your role within the emergency department (i.e. nurse, doctor, porter)?

How long have you worked in the ED?

Are there any dementia friendly initiatives used in your emergency department?

- What are these initiatives? How long have they been in place?

Have you received any staff training in relation to dementia?

- When? What did this entail? Did it include challenging behaviour? How useful was it?

Challenging behaviour

Have you experienced challenging behaviour from patients with dementia in the ED?

- YES: Can you tell us about these experiences?
  - What types of behaviours are exhibited?
  - How frequently do these instances occur?
  - Are these incidents reported?
  - Do you need other staff members to help? How many?
  - Did the patient need 1 to 1 care/support?
  - Are other patients impacted by this behaviour? In what way?
  - How are these situations de-escalated/ended?
  - How long do these situations last?
  - How long do these patients wait for a decision to be admitted or are they transferred to another area at the hospital such as a medical assessment unit?
  - Why do you think these situations occur? Any contributing factors/triggers?
  - Can you think of anything which may **prevent** these situations from occurring?
  - Can you think of anything which may **de-escalate** these situations once they are already happening?
- NO: If you have not had any experiences of this, why do you think these situations may occur? Any contributing factors/triggers?
  - Can you think of anything which may **prevent** these situations from occurring?
  - Can you think of anything which may **de-escalate** these situations once they are already happening?

Is there anything you can think of which could be changed about the ED environment in order to avoid challenging behaviour from patients with dementia?

- How can the organisation/staff/patients & their carers help?

For peer review only - http://bmjopen.bmj.com/site/about/guidelines.xhtml

Current management techniques

Have you witnessed any situations in the ED where sedation or restraint has been needed to manage challenging behaviour from patients with dementia?

Are there any assessment tools available within your ED to identify whether or not patients with dementia are likely to display challenging behaviour?

Intervention

Explain the aim to develop an intervention to prevent and/or de-escalate challenging behaviour from patients with dementia in the ED.

How do you feel about introducing this kind of intervention?

- Any possible challenges/best ways to do this?

Can you think of a way to **measure** the benefit of any new intervention?

- Physiological measures of the patient? Behavioural measures? Cognitive measures? Incident rates?

For peer review only

For peer review only - http://bmjopen.bmj.com/site/about/guidelines.xhtml

Checklist for Reporting Results of Internet E-Surveys (CHERRIES)*

Item category	Checklist item	Page no.	Description
Design	Study design	Pg 4	The target population was staff working in the emergency department of three UK Hospitals, responsible for responding to challenging behaviour. This was a convenience sample.
Ethics	Ethics approval	Pg 13	This study involves human participants and ethical approval was granted by the NHS Health Research Authority (20/HRA/0587) as well as the University of the West of England (UWE Bristol) Faculty of Health and Applied Sciences Research Ethics Committee (HAS-NAM-18-027). Participants gave informed consent to participate in the study before taking part.
	Informed consent	Pg 4	Informed consent for the survey was obtained from all those agreeing to complete a survey, with participant information displayed on the welcome page that the survey would take approximately 10 minutes to complete, that all responses were confidential and anonymous, and that data would be stored and analysed on password protected encrypted computers at UWE, accessed only by the study team, and deleted once the study findings are accepted for publication. A Privacy Notice was also available to download, and contact details were displayed for the research team. Consent was indicated when respondents clicked the 'I consent' button at the bottom of this page.<br><br>For interviews, informed consent was obtained through signed consent forms, after participants had been emailed a copy of the participant information sheet and privacy notice. Consent was then re-confirmed verbally at the start of the interview.
	Data protection	Pg 4	No personal information was collected. Survey data are stored on a secure server at UWE Bristol and the survey platform 'Qualtrics' (approved by UWE Bristol) for data security.
Development and pre-testing	Development and testing	Pg 4	The survey was designed using input from earlier research and review by the research team (healthcare professionals and researchers). A draft survey was piloted by members of the research team, as well as the patient and public involvement group.
Recruitment Process	Open vs closed Survey	Pg 4	This was a closed survey

For peer review only

	Contact mode	Pg 4	An email promoting the study (and including the survey link) was sent to all ED staff by local collaborators at each Trust and disseminated through bulletins and posters with a quick response (QR) code for the survey displayed in ED staff rooms at participating sites.
Recruitment process (cont'd)	Advertising the survey	Pg 4	As above
Survey administration	Web/email	Pg 4	This was a web-based survey, hosted by the survey platform 'Qualtrics'
	Context	Pg 4	Qualtrics is an online survey platform.
	Mandatory/voluntary	Pg 4	Voluntary.
	Incentives	N/A	N/A
	Time/date	Pg 4	Responses were collected between November 2020 and November 2021.

For peer review only

Item category	Checklist item	Page no.	Description
	Item randomisation	Appendix 1	No randomisation of items was used.
	Adaptive questioning	Appendix 1	Adaptive questioning (branched) was used. Relevant survey items were displayed based on previous responses.
	Number of items	Appendix 1	A maximum of four items were displayed on any one survey page.
	Number of screens	Appendix 1	The full survey was distributed over approximately 6 pages. A progress bar was shown at the top of the page, as the respondent was completing the survey.
	Completeness check	Appendix 1	All survey items were deemed to be mandatory, and respondents prompted to complete outstanding items before leaving the survey page on which the item was contained. Most items, except screener items and those items required for adaptive questioning included a 'Don't know/ none of the above' option.
	Review step	Appendix 1	Respondents were able to change their responses using a "Back" button at the bottom of each screen.
Response rates	Unique site visitor	N/A	N/A
	View rate	N/A	N/A
	Participation rate	N/A	N/A

For peer review only

Item category	Checklist item	Page no.	Description
	Completion rate	N/A	N/A
Preventing multiple entries from same individual	Cookies used	N/A	Not used
	IP check	N/A	Not used to maintain anonymity.
	Log file analysis	N/A	Not used.
	Registration	Pg 4	A login was not used - entry to the survey was via a web link emailed to eligible participants.
Analysis	Handling of incomplete questionnaires	Pg 4	Only completed questionnaires were included in the final dataset.
	Questionnaires with atypical timestamp	N/A	No respondents were removed from the survey for completing the items too quickly.
	Statistical correction	N/A	No weighting scheme was used for the analysis of results.

* Eysenbach G. Improving the quality of Web surveys: the Checklist for Reporting Results of Internet E-Surveys (CHERRIES). J Med Internet Res 2004;6:e34.

For peer review only

Supplementary Table 1: COREQ checklist

**Domain 1: Research team and reflexivity**		Location in manuscript (Section, page no.)
**Personal Characteristics**		
1. Interviewer/facilitator Which author/s conducted the interview or focus group?	LG, CL	Methods - 4
2. Credentials<br>What were the researcher's credentials? E.g. PhD, MD	BSc, PhD	Title Page
3. Occupation<br>What was their occupation at the time of the study?	LG: Senior Research Fellow in Emergency Care<br><br>CL: Research Associate	Title Page
4. Gender Was the researcher male or female?	Female	N/A
5. Experience and training<br>What experience or training did the researcher have?	Experienced qualitative researchers.	Methods - 4
**Relationship with participants**		
6. Relationship established<br>Was a relationship established prior to study commencement?	No	N/A
7. Participant knowledge of the interviewer<br>What did the participants know about the researcher? e.g. personal goals, reasons for doing the research	Participants were briefed on the purpose of the study and were aware that ethical approval had been granted. Participants reviewed the participant information documentation prior to giving their written informed consent to be involved.	Methods - 4
8. Interviewer characteristics What characteristics were reported about the interviewer/facilitator? e.g. Bias, assumptions, reasons and interests in the research topic	No interviewer-related biases identified.	N/A
**Domain 2: study design**		
**Theoretical framework**		
9. Methodological orientation and Theory<br>What methodological orientation was stated to underpin the study? e.g. grounded theory, discourse analysis, ethnography, phenomenology, content analysis	Thematic analysis	Methods – 5
Participant selection		
10. Sampling How were participants selected? e.g.	Convenience sampling was used: an email promoting the study (and	Methods - 4

For peer review only

	including the survey link) was sent to all ED staff by local collaborators at each Trust and disseminated through bulletins and posters	
11. Method of approach How were participants approached? e.g. face-to-face, telephone, mail, email	As above	Methods - 4
12. Sample size How many participants were in the study?	13 interview participants	Results - 5
13. Non-participation How many people refused to participate or dropped out? Reasons?	Of the first 13 respondents that were invited for a semi-structured interview, all gave informed consent and completed the interview. There were no participants who subsequently refused to participate, withdrew consent or dropped out.	N/A
**Setting**		
14. Setting of data collection<br>Where was the data collected? e.g. home, clinic, workplace	Data was collected via telephone and audio-recorded using Skype for business	Methods - 4
15. Presence of non-participants<br>Was anyone else present besides the participants and researchers?	No	N/A
16. Description of sample<br>What are the important characteristics of the sample? e.g. demographic data, date	There were mostly female (n = 10, 77%). Length of experience working in ED ranged from less than 3 years to over 10 years. Data were held between October 2020 and November 2021	Results – 5-6
**Data collection**		
17. Interview guide Were questions, prompts, guides provided by the authors? Was it pilot tested?	Interviews were semi-structured using a topic guide; the topic guide was developed and reviewed by the study team. This topic guide was then reviewed and refined by the Patient and Public Involvement contributors.	Methods - 4
18. Repeat interviews<br>Were repeat interviews carried out? If yes, how many?	No	N/A

For peer review only

19. Audio/visual recording<br>Did the research use audio or visual recording to collect the data?	The semi-structured interviews were audio recorded using skype for business.	Methods - 4
20. Field notes<br>Were field notes made during and/or after the interview or focus group?	No additional field notes were made.	N/A
21. Duration What was the duration of the interviews or focus group?	The interviews lasted an average of 45 minutes	Methods - 4
22. Data saturation<br>Was data saturation discussed?	We used information power	Methods - 4
23. Transcripts returned Were transcripts returned to participants for comment and/or correction?	No	N/A
**Domain 3: analysis and findings**		
**Data analysis**		
24. Number of data coders<br>How many data coders coded the data?	Two – LG and CL	Methods - 5
25. Description of the coding tree<br>Did authors provide a description of the coding tree?	Coding described in methods section.	Methods - 5
26. Derivation of themes<br>Were themes identified in advance or derived from the data?	Themes were derived from the data	Methods - 5
27. Software What software, if applicable, was used to manage the data?	NVivo 12	Methods - 5
28. Participant checking<br>Did participants provide feedback on the findings?	No	N/A
**Reporting**		
29. Quotations presented Were participant quotations presented to illustrate the themes / findings? Was each<br>quotation identified? e.g. participant number	Yes	Results – 5-11
30. Data and findings consistent<br>Was there consistency between the data presented and the findings?	Yes	N/A
31. Clarity of major themes<br>Were major themes clearly presented in the findings?	Yes	Results 5-11
32. Clarity of minor themes<br>Is there a description of diverse cases or discussion of minor themes?	Yes	Results 5-11

For peer review only

How do Emergency Department staff respond to behaviour that challenges displayed by people living with dementia? A mixed-methods study

Journal:	*BMJ Open*
Manuscript ID	bmjopen-2023-075022.R1
Article Type:	Original research
Date Submitted by the Author:	13-Jul-2023
Complete List of Authors:	Goodwin, Laura; University of the West of England<br>Liddiard, Cathy; University of the West of England<br>Manning, Sera; University of the West of England<br>Benger, Jonathan; University of the West of England<br>Carlton, Edward; North Bristol NHS Trust<br>Cheston, Richard; University of the West of England<br>Hoskins, Rebecca; University Hospitals Bristol and Weston NHS Foundation Trust<br>Taylor, Hazel; University Hospitals Bristol NHS Foundation Trust, Research Design Service<br>Voss, Sarah; University of the West of England
**Primary Subject Heading**:	Emergency medicine
Secondary Subject Heading:	Communication, Health services research
Keywords:	Dementia, ACCIDENT & EMERGENCY MEDICINE, Hospitals, Public, Aging

SCHOLARONE™
Manuscripts

I, the Submitting Author has the right to grant and does grant on behalf of all authors of the Work (as defined in the below author licence), an exclusive licence and/or a non-exclusive licence for contributions from authors who are: i) UK Crown employees; ii) where BMJ has agreed a CC-BY licence shall apply, and/or iii) in accordance with the terms applicable for US Federal Government officers or employees acting as part of their official duties; on a worldwide, perpetual, irrevocable, royalty-free basis to BMJ Publishing Group Ltd ("BMJ") its licensees and where the relevant Journal is co-owned by BMJ to the co-owners of the Journal, to publish the Work in this journal and any other BMJ products and to exploit all rights, as set out in our licence.

The Submitting Author accepts and understands that any supply made under these terms is made by BMJ to the Submitting Author unless you are acting as an employee on behalf of your employer or a postgraduate student of an affiliated institution which is paying any applicable article publishing charge ("APC") for Open Access articles. Where the Submitting Author wishes to make the Work available on an Open Access basis (and intends to pay the relevant APC), the terms of reuse of such Open Access shall be governed by a Creative Commons licence – details of these licences and which Creative Commons licence will apply to this Work are set out in our licence referred to above.

Other than as permitted in any relevant BMJ Author's Self Archiving Policies, I confirm this Work has not been accepted for publication elsewhere, is not being considered for publication elsewhere and does not duplicate material already published. I confirm all authors consent to publication of this Work and authorise the granting of this licence.

For peer review only

How do Emergency Department staff respond to behaviour that challenges displayed by people living with dementia? A mixed-methods study

Authors:
Goodwin, L.[1], Liddiard, C.[1], Manning, S.,[1] Benger, J.[1], Carlton, E.[2], Cheston, R.[1], Hoskins, R.[3], Taylor, H.[3], & Voss, S.[1]

Institutions:
[1] University of the West of England, Bristol
[2] North Bristol NHS Trust
[3] University Hospitals Bristol and Weston NHS Foundation Trust

Corresponding author:
Laura Goodwin – laura.goodwin@uwe.ac.uk

Keywords:
Dementia, Emergency Service, Hospital, Behavioural Symptoms, Aggression

List of abbreviations:
BtC – Behaviour that challenges
ED – Emergency department

Word count:
4688

For peer review only

ABSTRACT

Objectives: To investigate staff experiences of, and approaches to behaviour that challenges displayed by patients with dementia in the Emergency Department. Behaviour that challenges is defined as 'actions that detract from the well-being of individuals due to the physical or psychological distress they cause within the settings they are performed', and can take many forms including aggressive physical actions, shouting and verbal aggression and non-aggressive behaviour including repetitive questioning, inappropriate exposure and resistance to care.

Design: Mixed-methods study consisting of an online survey and semi-structured telephone interviews. Quantitative data were analysed and presented using descriptive statistics. Qualitative data were analysed thematically.

Setting: The Emergency Departments of three NHS Hospital Trusts in Southwest England.

Participants: Multidisciplinary NHS staff working in the Emergency Department.

Results: Fifty-two online survey responses and 13 telephone interviews were analysed. Most (24/36, 67%) survey respondents reported that they had received general training in relation to dementia in the last 2-5 years, however less than a fifth (4/23, 17%) had received any ED-specific dementia training. All (48/48) felt that behaviour that challenges could potentially be prevented, though resource constraints and practice variation were identified. Four main themes emerged from the qualitative data: (1) The "perfect storm" of the ED; (2) Behaviour that challenges is preventable with the right resources; (3) Improvisation; (4) Requirement for approaches that are specific to the Emergency Department.

Conclusions: The findings of this study suggest that Emergency Department staff do not feel that they are prepared to respond effectively to behaviour that challenges displayed by people living with dementia. Future work could adapt or develop an intervention to support Emergency Department staff in responding to behaviour that challenges.

STRENGTHS AND LIMITATIONS OF THIS STUDY

- To the best of our knowledge, this is the first study to focus specifically on staff experiences of responding to behaviour that challenges associated with dementia in United Kingdom Emergency Departments.
- A mixed-methods study design including survey and qualitative interviews provided both an overview and more detailed description of staff experiences.
- The survey had a relatively low response rate and the research was confined to one geographical area which may limited the representativeness of our findings.

FUNDING STATEMENT

The study was funded by the University Hospitals Bristol and Weston NHS Foundation Trust Above and Beyond (Charitable Trustees of UH Bristol) and UH Bristol Research Capability Funding (RCF) Grants awarded through the Research Funding Committee (2019-Spr-03).

COMPETING INTERESTS

None declared

For peer review only - http://bmjopen.bmj.com/site/about/guidelines.xhtml

BACKGROUND

In the United Kingdom (UK), there are around 944,000 people living with dementia, and this is estimated to increase to more than 1.6 million by 2050.(1) Challenging behaviour, also referred to by the Alzheimer Society and others as ‘behaviour that challenges’ (BtC),(2) is commonly associated with dementia and has been defined as “actions that detract from the well-being of individuals due to the physical or psychological distress they cause within the settings they are performed”.(3) BtC can take many forms including aggressive physical actions (e.g., kicking, biting), shouting and verbal aggression and non-aggressive behaviour including repetitive questioning, inappropriate exposure and resistance to care. This behaviour often stems from an unmet need – for instance to reduce distress or frustration or to maintain a sense of wellbeing. Both the person living with dementia and the people around them are likely to be impacted by their behaviour.(4)

An increase in the number of people living with dementia is reflected in emergency hospital admissions. In England there was a rise of 35% in emergency admissions for people with dementia during 2017-18, compared with 2012-13.(5) More than half of the people who had a diagnosis of dementia in 2017-18 were admitted to hospital as an emergency at least once in that year.(5)

In a busy Emergency Department (ED), the physical and medical aspects of care such as completing observations and administering medication may become prioritised over less technical elements (for example helping with toileting or food/drink),(6) and this may result in unmet needs for older people living with dementia.(7) For instance, the loud and often crowded environment of the ED can result in sensory overload for people living with dementia,(8,9) and may lead to dehydration, ineffective pain management exacerbating other unmet needs with an increased risk of BtC.(10–12)

Behaviour that challenges can be difficult to respond to for ED staff, who need to ensure safety and prompt treatment for all patients. In a retrospective audit by Tropea et al. (2017),(13) 39% of people living with dementia attending an ED in Australia exhibited behaviour that challenges, with aggression being the most common (35%). Despite the importance of dementia training being recognised by Royal College of Emergency Medicine,(14) there are no standardised processes for managing patients living with dementia or cognitive impairment in UK EDs. Without training and resources to prevent or respond to challenging behaviour associated with dementia, ED staff have previously relied on sedating or restraining patients, which is often distressing for patients, family members and staff and likely to lead to adverse outcomes.(13,15)

Various approaches are available to hospital staff for responding to challenging behaviour of people living with dementia: creating a calm, quiet physical environment;(16) sensory stimulation such as light and music therapy;(17) distraction activities such as video, music boxes or dolls;(18) accessing ‘hospital ready’ information about the patient and their wishes.(19,20) However, there is little research on the effectiveness of prevention or de-escalation interventions in EDs,(15) where there may be unique challenges to implementing some of these approaches. This means that there is no standardised use of these approaches across EDs in the UK. Therefore, this study aimed to investigate UK National Health Service (NHS) staff experiences of responding to BtC, associated with dementia, in the ED. The aims of this project were:

1. To explore staff experiences of, and approaches to behaviour that challenges associated with dementia in the ED environment.

For peer review only - http://bmjopen.bmj.com/site/about/guidelines.xhtml

2. To explore ED staff opinions of using de-escalation techniques within an ED setting and any perceived barriers and facilitators to introducing new interventions.

METHODS

Setting

The setting was the EDs in three NHS Hospital Trusts in the Southwest of England: University Hospitals Bristol and Weston NHS Foundation Trust (UHBW), North Bristol NHS Foundation Trust (NBT), and Royal United Hospitals Bath NHS Foundation Trust (RUH). ED attendance at each site is as follows: over 90,000 people a year at NBT; over 100,000 people a year at UHBW; over 70,000 a year at RUH.(21) There are estimated to be more than 2,574 people in Bath and North East Somerset currently living with dementia, and 4,500 people living with dementia in Bristol.(22,23) All participating Trusts currently use the 'This is Me' (20) document for attending patients who are known to be living with dementia.

Patient and public involvement (PPI)

PPI involvement was gained from a local support group for people living with dementia and their carers in Bristol, who have experience of attending local EDs as/with a person living with dementia. The group provided feedback on the study aims, design and recruitment, and supported the research team in designing all patient-facing documents.

Study design

Mixed-methods study consisting of an online survey and semi-structured interviews. Both the survey and interviews were open to all staff working in the three NHS Hospital Trust EDs above. Convenience sampling was used: an email promoting the study (and including the survey link) was sent to all ED staff by local collaborators at each Trust and disseminated through bulletins and posters with a quick response (QR) code for the survey displayed in ED staff rooms at participating sites. Recruitment adverts included an offer of a £10 voucher for those who completed an interview.

The survey was hosted on the 'Qualtrics' platform (Provo, Utah) and accessed through the disseminated links and QR codes. Participants were required to confirm consent electronically before the first survey question was presented. As the survey was exploratory, a sample size calculation was not required.

Eligible participants were invited to contact the lead author to take part in an interview. Potential interview participants received a participant information sheet, privacy notice and consent form via email. Verbal confirmation of consent was audio-recorded at the start of the telephone interview. We aimed to recruit 10-15 participants, guided by information power with a narrow aim and specific study population.(24)

Data collection

Survey

The open online survey (Supplemental file 1) was developed by the study team from the findings of a literature review on responding to BtC in people with dementia in the ED, (25) in collaboration with ED colleagues. It was then reviewed by members of our PPI group, and piloted for usability and technical functionality by members of the study team. The survey was open between November 2020 and November 2021. A participant information sheet, privacy notice and consent form were integrated into the first page of the survey. The survey contained questions about the extent and nature of the issues associated with caring for patients with

For peer review only - http://bmjopen.bmj.com/site/about/guidelines.xhtml

dementia whose behaviour may be challenging in an ED setting. Questions with open free-text responses were included alongside questions with Likert scales and multiple-choice responses.

Interviews

The participant information sheet and consent form were developed in collaboration with the study team and our PPI group. The interview topic guide (Supplemental file 2) was drafted by the study team, based on existing literature and the study team's expertise. It then was reviewed by our PPI group, and piloted with clinical colleagues. Semi-structured interviews were used to investigate staff experiences of responding to BtC associated with dementia in the ED and any prevention/de-escalation techniques used. A single interview was held with each participant, which was conducted via telephone at a time convenient for the participant. Interviews were held between October 2020 and November 2021 by experienced qualitative researchers (LG and CL) and audio-recorded via Skype for Business. Interviews lasted an average of 45 minutes. Audio files were transcribed verbatim.

Analysis

Survey data were exported from Qualtrics to a Microsoft Excel spreadsheet. Quantitative data were analysed and presented using descriptive statistics. Free-text data were summarised and categorised.

Interview transcripts were coded and analysed by CL and LG, supported by NVivo 12 software. Data were analysed thematically through a well-established iterative process of data reduction, constant comparison, organisation and understanding.(26) The researchers read the transcripts several times and then coded selections of text to represent instances of a concept. Codes were then reviewed in terms of their relationship to other codes and combined to create more developed themes. From this analysis, distinctions could be made between the different levels of themes.

RESULTS

Participant characteristics

A total of 52 responses to the survey were received from ED staff (Table 1). Thirteen interviews were conducted (Table 2). The largest proportion of survey participants (22/52, 42%) were doctors, whilst the majority of telephone interviewees (8/13, 62%) were nurses. We did not collect data on gender for the survey, but the majority of telephone interviewees (10/13, 77%) were female.

Table 1. Survey respondent characteristics (n=52)

**Characteristic**		**No. (%) of respondents**
Role		
	Doctor	22 (42%)
	Nurse	19 (37%)
	Admin staff	1 (2%)
	Porter	1 (2%)
	Other	9 (17%)
Approximate experience working in ED (years)		
	<1	6 (12%)
	1-2	13 (25%)
	3-5	8 (15%)

For peer review only - http://bmjopen.bmj.com/site/about/guidelines.xhtml

For peer review only

	6-10	9 (17%)
	11-15	8 (15%)
	16-20	5 (10%)
	21+	3 (6%)

Table 2: Interview participant characteristics (n=13)

**Characteristic**		**No. (%) of respondents**
Role		
	Doctor	4 (31%)
	Nurse	8 (62%)
	Other	1 (7%)
Gender		
	Female	10 (77%)
	Male	3 (23%)
Approximate experience working in ED (years)		
	≤3	4 (31%)
	4-6	5 (39%)
	7-9	2 (15%)
	10+	2 (15%)

Four main themes were identified from the qualitative data: (1) The "perfect storm" of the ED; (2) Behaviour that challenges is preventable with the right resources; (3) Improvisation; (4) Requirement for approaches that are specific to the Emergency Department. These are reported in narrative form, supported by quotes, and woven into the quantitative results below.

Theme 1: The "perfect storm" of the ED

The significant challenges that the ED setting presents to people with dementia was described by all interview participants. Survey data indicated that almost all (49/52, 94%) respondents had experienced BtC in the ED from patients living with dementia. Telephone interviewees perceived the 'perfect storm' of the ED to be made up of triggers (physiological and environmental), the ED's clinical approach to patients, and a lack of resources.

The patient being in pain was most commonly ranked as the number one trigger for BtC by survey participants (14/46, 30%). During telephone interviews however, most participants described the hospital environment itself as the dominant triggering factor for BtC due to an absence of familiar faces and routine. Most participants reported that there to were factors unique to the ED (in comparison to other hospital settings) which compounded patients' unmet needs such as: constant bright lighting; the busy nature of the department; long waits; open areas that pose a risk of absconding; staff having less time to get to know their patient.

> It's noisy, it's bustling, it's confusing, you [the patient] often feel isolated, there are people walking past you all the time looking at you. There are strangers coming in and out. I mean everything in ED plays against dementia. You know, for those of us that are used to the chaos of the place it can still be challenging on a bad day, but to somebody with dementia it must be a nightmare. It's got everything going against it. (P011)

For peer review only - http://bmjopen.bmj.com/site/about/guidelines.xhtml

Inadequate information transfer was also highlighted as a resource issue for effectively managing BtC in the ED. Interview participants reported incomplete handovers from ambulance staff, absent personalised care plans, and less chance of family/friends being able to communicate the patient's needs, due to restrictions on visitors since related to the Covid-19 pandemic.

Some participants described how the primary function of the ED is to focus on the patient's presenting clinical condition (for example stemming the bleeding from a fall, or providing treatment for blood pressure), which meant that they were often unable to remove the environmental triggers in the ED for patients living with dementia, as they were necessary for providing safe medical care.

> Everybody needs to back off and leave them for a while because I think any form of intervention only escalates the situation. The trouble is, of course, we are under time pressures and our focus is on getting the injury sorted. (P011)

Within this 'perfect storm', telephone interviewees recalled numerous experiences of BtC in the ED, with many reporting instances occurring on every shift. Survey data reflected the telephone interviews, where the majority of participants (36/46, 78%) reported that BtC occurred on a weekly basis in their ED. The most common types of behaviour reported by survey participants included 'confusion' (45/46; 98%), 'wandering' (43/46; 94%), 'agitation', (43/46; 94%) and 'verbal aggression' (42/46; 91%). Telephone interview participants described instances of all of these behaviours within their ED, and reported concerns regarding patient safety and staff resource.

> The most frequent experience that happens with a patient with dementia is that they come into the emergency department and they wander, and unfortunately we don't have a dedicated member of staff to stay with them. So, it's scary because as a nurse you feel that they are in danger. (P007)

Some behaviours were reported as being more challenging in an ED environment, and participants expressed anxiety around gaps in dementia care in the ED.

> I don't think there's any areas that are 100% safe. I know you can't be with dementia but it's quite risky in ED. But then they're only supposed to be there for four hours and then go onto a ward. But that obviously doesn't always happen with, you know, lack of beds. (P006)

Staff interviewed via telephone reported that prevention and de-escalation of BtC could be difficult to maintain in the ED environment and that, if responded to ineffectively then behaviours found to be challenging by staff could continue throughout the patient's entire ED stay.

> Nine times out of ten [the BtC lasts] until they get to a ward, or until they are picked up by transport. (P001)

> I would say there are nights where we don't really get people settled at any point. (P012)

Some of these interviewees described how the challenge of managing BtC worsened at night, as people living with dementia often experienced sleep deprivation and were unable to settle in the

For peer review only - http://bmjopen.bmj.com/site/about/guidelines.xhtml

ED environment. One reported the rate of falls to be higher at night, and noted difficulties escalating patients or getting support from dementia teams during this time.

Theme 2: Behaviour that challenges is preventable with the right resources

Survey respondents indicated they felt that instances of BtC could either be prevented (13/48 30%) or sometimes prevented (35/48, 73%) in the ED. One-to-one care from staff or a carer was ranked as the intervention most likely to prevent BtC (18/44, 41%), followed by rapid assessment of the patient (10/44, 23%). For de-escalating BtC, one-to-one care was also the highest-ranking technique (25/44, 57%), followed by the provision of a distraction (7/44, 16%) or a quieter environment (7/44, 16%). However, participants in both the survey and interviews highlighted the lack of resources available to provide these types of interventions, noting concerns regarding crowded EDs, understaffing and long ambulance wait times.

> It's the endless resources isn't it, that we don't have? If there were a team of Dementia Nurses in the hospital that you know, Healthcare Assistants even, that could just come and sit with the patients, engage them, in looking at that magazine, listening to that music, you know, or whatever it is, if it's just sitting and chatting with them. (P009)

Despite a common acknowledgment of the unique challenges of the ED for people living with dementia, less than a fifth (4/23, 17%) of survey respondents had received dementia training that was specific to the ED context. None of the telephone interviewees reported having had any ED-specific dementia training.

While most survey respondents (24/36, 67%) reported that they had received general training in relation to dementia in the last 2-5 years, a large proportion (11/23, 48%) found it only 'moderately useful'. This was reflected in interview data, where general dementia training was given mixed reviews in terms of its usefulness.

Most telephone interviewees could recall generic dementia-friendly initiatives that had been implemented in their ED. One of the initiatives, music, was enthusiastically highlighted as being particularly helpful to patients, although frustration was expressed at how quickly equipment would go missing. Other de-escalation techniques were also perceived as useful to staff and patients, but telephone interview participants noted that such techniques often needed an investment of time and staff to facilitate them, which was not always possible. Indeed, limitations (most often linked to resource issues) were described for most of the initiatives described positively by staff (Table 3).

Table 3. Existing dementia-friendly initiatives recalled by interview participants.

Initiative	Limitation(s)
Reminders of the past: designing corridors with pictures of past-times and/or old films played on a TV	• They become out of date.
Colouring Books	• Not always in stock.

For peer review only

Box of distractions	• Requires space/tables to use.<br>• Can require a staff member to facilitate their use.<br>• Not available during Covid-19 as has to go through infection control.
Providing food and drink	• Short-term solution<br>• Hot food not always available in ED.<br>• White plates can cause confusion with some (white) foods.
'This is Me' (20) or similar: document containing information about the patient's needs and wishes	• Patient does not always come in with it.<br>• Specific paperwork required to create one is not in stock<br>• No time to create one in ED setting.<br>• Suggestions within it may not be appropriate for the ED setting.
Music	• Equipment went missing.
Family member/carer	• Covid-19 restrictions reduced this.
Individual cubicles	• Not always safe, as unable to see the patient.<br>• Many cubicles not available as used for patients with Covid-19 or other infectious conditions

The majority of survey respondents (30/45, 67%), felt that there should be a separate assessment area of the ED for patients with dementia. Free-text comments added that the ideal space should be quiet, where the lights can be dimmed and attended by staff trained in dementia. From respondents that did not think there should be a separate assessment area (33% 15/45), free-text comments showed support for this type of area in an "ideal world" but reported that the logistical and resource challenges this would present to enable patients to be safe there was likely to render the initiative unfeasible.

Theme 3: Improvisation

While most survey respondents (23/36, 64%) reported that they covered information about how to manage BtC in their general dementia training, there was variation in responses to BtC by the ED staff surveyed. The most used approaches to resolving or reducing the impact of BtC were distraction techniques (37/46, 81% reported using them 'always' or 'most of the time'), and the use of family and/or carers (20/46, 43% reported using them 'always' or 'most of the time').

Some telephone interviewees reported using their own individual approaches to preventing and de-escalating BtC, learnt from other professional roles or their personal life. One described 'messing-up' sheets and asking patients to fold them, or offering bandages to squeeze whilst carrying out a procedure to keep their hands occupied.

For peer review only - http://bmjopen.bmj.com/site/about/guidelines.xhtml

> Everyone uses their own sort of techniques. No-one's really said you can or can't do this, but you just have to improvise with what you've got and it's difficult when you haven't got a lot to help you. (P011)

Telephone interview participants reported that an inconsistent approach to managing BtC often resulted in ineffective or non-sustained management of behaviour within the ED, and that this became particularly problematic when it led to patients resisting medical care. Indeed, the majority of survey respondents (87%, 40/46) reported that they had experienced physical resistance to care when supporting people living with dementia in the ED. Participants interviews via telephone reported that this often meant that procedures were either minimised or postponed, or described some kind of 'battle' with the patient to complete the procedure; causing distress for both patient and staff.

> I had a lady yesterday, who…needed a Lateral Flow test. So, you know, to go and stick a thing up her nose, she didn't want that done. And it was a horrible thing to have to do and, in the end, I gave up with it, because it was just distressing her too much... But yeah, she was grabbing and pinching [me] and you know, she, she was quite up for giving you a hearty thump at the end of it. (P009)

When asked directly about the use of physical restraint as a response to BtC in the ED, most survey respondents (36/46, 78%) reported that they never used it. However, there seemed to be some differences in how the word 'restraint' was interpreted by telephone interview participants, which often led to contradictory accounts during interviews. While most telephone interviewees said that restraint was only very rarely used, when questioned directly, many described using or witnessing some form of restraint frequently (to administer care) when talking more generally about their experiences of managing BtC in the ED.

> I've not seen anyone have to be physically restrained or anything like that. I think we try to kind of implement things in place before anything like that would need to happen. (P002)

> For simple things like taking a blood test…you'll often hold the patient's hand down, or arm down, and they can't consent. (P005)

There was also a contradiction between survey and interview responses when reporting the use of pharmaceutical measures to manage BtC in the ED. Telephone interviewees were unified in their perception that the use of pharmaceutical measures to reduce BtC in the ED was very rare, whilst the majority (38/46, 83%) of survey respondents reported that this approach was 'sometimes' used. Again, there seemed to be some differences in the interpretation of the wording, which meant that mild sedation to complete a medical procedure was not always viewed as a pharmaceutical measure to manage BtC.

Some telephone interview participants described an accelerated approach to completing medical assessments/procedures to minimise the chance of the patient showing physical resistance to care, even if this meant completing tasks without explicit consent.

> It's horrendous, but you just do things. You almost don't ask, if you're doing these things… I might say, 'oh I'm going to exam your eyes, that's okay?' But you just roll off the natural things, and the nurses do them, and you're doing it out of best interest, but sometimes it's easier to be like 'oh, they don't

For peer review only - http://bmjopen.bmj.com/site/about/guidelines.xhtml

> necessarily understand' and you just crack on and do it... You're just trying to be, like, she probably won't understand the blood test and things, but I think she really needs it, she might have a raging infection, we need to do this. (P005)

Theme 4: Requirement for approaches that are specific to the Emergency Department

There was overwhelming support from telephone interview participants regarding the potential introduction of an ED-specific intervention to support staff in managing BtC. In fact, one participant had developed their own dementia training package, specific to the ED. This training aimed to encourage staff to consider the potential trigger(s) of the BtC (i.e., toileting needs, discomfort/pain) and ways to minimise these triggers, whilst the patient was in the ED.

A number of participants put forward the idea of a simple check-list or escalation toolkit for care, which could use a *'step-wise approach that will guide you in both recognising and dealing with these behaviours'* (P004). Participants said that a formal tool would increase the consistency of approach to BtC across the ED and provide something to follow when behaviour was escalating. One interviewee described their vision of what an 'escalation tool' could look like:

> If it was like a scale of one to ten, the first thing is make sure they're not in pain and then the second thing is do they need the toilet, almost like a tick box of things you need to consider, and then you work your way up…from basic things to, kind of, more difficult things to implement. Then you could tick them off almost - what you've done and at what point it de-escalated. So then it would potentially help with their care in the future when they're on the wards or if they came back into ED. (P002)

Another suggested that a new intervention should give more emphasis to preventing triggers for BtC, rather than focusing solely on de-escalation.

> I guess the bit we don't talk about is preventing those behaviours happening in the first place, and how to make our department a place that prevents things escalating…We wait for it to escalate and then we think about, is this patient comfortable? Are they in a quiet enough environment? Do they have food and drink? (P012)

Other suggestions included better identification of patients who may be living with dementia, specialist ED-specific training for staff, and improved co-ordination with other services and organisations. For example, participants reported that there could be better handover from care-homes and ambulance services, including multi-service utilisation of an electronic version of the 'This is Me'(20) document, or arranging for home-comforts to be brought to the ED with the patient.

> We don't think enough about them having something from their home or from their care home, like a blanket or something that they find familiar, that has the right smell. (P007)

There were some potential barriers put forward by ED staff when implementing a new intervention. Telephone interview participants advised that any new initiative should not need too much space or time, be complex, or *'another piece of paperwork to fill out'* (P012). They also warned that measuring the impact of any new intervention may be challenging.

For peer review only - http://bmjopen.bmj.com/site/about/guidelines.xhtml

Facilitators to a new intervention, put forward by telephone interview participants, included buy-in from key stakeholders and emphasis on how the intervention would help patients. One interviewee suggested the use of face-to-face study days to explain the need for a new tool, whilst another emphasised the need for any information to be distributed to all ED staff who would come into contact with patients (i.e. including porters, receptionists, security guards and health care assistants).

DISCUSSION

To the best of our knowledge, this is the first study to specifically focus on staff experiences of responding to behaviour that challenges (BtC) in the Emergency Department (ED) environment in the UK. A mixed-methods study design combining survey and qualitative interviews provided both a wide overview as well as a rich description of staff experiences. Important weaknesses of our study include the relatively low response rate and the fact that the research was confined to one geographical area which will have limited the representativeness of our findings. We used a convenience sample of volunteers, and so the experiences and views on which our research is founded may not be representative of all ED staff.

We found several challenges associated with responding to BtC associated with dementia in the ED. These included the concept of the ED as a ‘perfect storm’ for behavioural triggers, a lack of resources to effectively prevent and de-escalate BtC within the ED, and inconsistent approaches to managing BtC, leading to escalation of behaviour. Staff were positive about the idea of introducing ED-specific interventions to prevent/de-escalate BtC and offered a number of suggestions for what this might include.

The description of the ED as a ‘perfect storm’ is well supported in the literature regarding dementia.(27) Our findings suggest a lack of ED-specific training and resources. While a number of different interventions have been found to be helpful in the general hospital setting, the effectiveness of these approaches in the ED is unknown due to a lack of research.(15) Participants in our study noted that a lack of ED-specific training and resources led to variation in the techniques used by staff to respond to and de-escalate BtC, which in turn led to ineffective management; staff were left to improvise with the resources they had, often attempting to intervene once behaviours had already escalated substantially.

In line with previous literature, our findings suggest that ineffective management of BtC sometimes resulted in physical and pharmacological restraint being used by staff in the ED.(13) However, there seemed to be differences in the interpretation of words such as ‘restraint’ and ‘sedation’ by participants; some staff did not perceive physical measures used to facilitate minor procedures as formal restraint. Similar findings were noted in a qualitative study of Australian emergency nurses who did not perceive manual restraint for minor injuries care as “formal” restraint.(28)

Our findings reflect the high frequency of BtC in the ED reported in other literature.(13) While staff felt that that some incidents of BtC could be prevented, this was mainly through resource-intense approaches (i.e. 1:1 care or a dedicated space for triage and assessment), which participants felt was unlikely to prove realistic in the context of resource limitations. The under-use of family and friends was noted in research prior to Covid-19,(29) and is likely to have been exacerbated during and after the pandemic.

For peer review only - http://bmjopen.bmj.com/site/about/guidelines.xhtml

Participants in our study expressed support for a specific checklist or flowchart approach to guide care and behaviour management in the ED. These have been used successfully in a variety of healthcare settings to act as an aid to decision-making or memory recall, including responding to BtC for people living with dementia in residential care.(30,31)

This study has important implications for practice. Findings suggest the need for regular site-wide dementia education for all staff, including information specific to the ED environment and better education on the approaches that can be used to prevent and de-escalate BtC. They also highlight the need for evidence-based resources to support this population of patients in the ED, and greater collaboration with family members and carers, to prevent unnecessary use of sedation and restraint.

CONCLUSION

The findings of this study suggest that Emergency Department staff do not feel they are prepared to effectively respond to BtC in the ED due to the unique combination of triggers presented by the environment, and the lack of resources available in this setting. This is an increasing issue, due to the rising numbers of ED admissions for people living with dementia. Future work could adapt or develop an intervention to effectively support ED staff in managing BtC associated with dementia.

For peer review only

STATEMENTS

Acknowledgments

We would like to thank the ED staff who took part in interviews for this research, as well as those who promoted this study.

Patient and Public Involvement

Patients and/or the public were involved in the design, or conduct, or reporting, or dissemination plans of this research. Refer to the Methods section for further details.

Patient consent for publication

Not required

Ethics approval

This study involves human participants and ethical approval was granted by the NHS Health Research Authority (20/HRA/0587) as well as the University of the West of England (UWE Bristol) Faculty of Health and Applied Sciences Research Ethics Committee (HAS-NAM-18-027). Participants gave informed consent to participate in the study before taking part.

Provenance and peer review

Not commissioned; externally peer reviewed.

Contributorship statement

SV, SM, EC, RC, RH, HT and JB participated in study conception, design and delivery. LG and CL participated in study coordination, performed and coded the interviews, analysed the data and

For peer review only - http://bmjopen.bmj.com/site/about/guidelines.xhtml

drafted the manuscript. All authors were responsible for the critical revision of the manuscript for publication and approved the final version. SV acts as guarantor.

Competing interests

None declared

Funding

The study was funded by the University Hospitals Bristol and Weston NHS Foundation Trust Above and Beyond (Charitable Trustees of UH Bristol) and UH Bristol Research Capability Funding (RCF) Grants awarded through the Research Funding Committee (2019-Spr-03).
2019-Spr-03

Data sharing statement

Data are available upon reasonable request.

For peer review only

REFERENCES

1. Alzheimer's Research UK. Dementia Statistics Hub: Prevalence projections in the UK [online]. 2022. https://dementiastatistics.org/statistics/prevalence-projections-in-the-uk-2/ (accessed 17 March 2023).

2. Alzheimer's Society. How does dementia change a person's behaviour? [online]. 2021. https://www.alzheimers.org.uk/about-dementia/symptoms-and-diagnosis/symptoms/behaviour-changes#content-start (accessed 17 March 2023).

3. James IA, Jackman L. Understanding behaviour in dementia that challenges: A guide to assessment and treatment. London: Jessica Kingsley Publishers 2017.

4. Cohen-Mansfield J. Agitated behaviors in the elderly: II. Preliminary results in the cognitively deteriorated. J Am Geriatr Soc. 1986;34(10):722–7.

5. Alzheimer's Society. Analysis of NHS England's Hospital Episode Statistics dataset 2012/13 to 2017/18. [Online]. 2020. www.alzheimers.org.uk/sites/default/files/2020-01/Hospital%20Admissions%202012-18%20for%20people%20with%20dementia%20Alzheimer%27s%20Society%202020.pdf (accessed 17 March 2023).

6. Mollaoğlu M, Çelik P. Evaluation of emergency department nursing services and patient satisfaction of services. J Clin Nurs. 2016;25(19–20):2778–85.

7. Parke B, Hunter KF, Marck PB. A novel visual method for studying complex health transitions for older people living with dementia. Int J Qual Methods. 2015;14(4):1609406915614150.

8. Silwanowicz RM, Maust DT, Seyfried LS, et al. Management of older adults with dementia who present to emergency services with neuropsychiatric symptoms. Int J Geriatr Psychiatry. 2017;32(12):1233–40.

9. Clevenger CK, Chu TA, Yang Z, et al. Clinical care of persons with dementia in the emergency department: a review of the literature and agenda for research. J Am Geriatr Soc. 2012;60(9):1742–8.

10. Burgstaller M, Mayer H, Schiess C, et al. Experiences and needs of relatives of people with dementia in acute hospitals—A meta-synthesis of qualitative studies. J Clin Nurs. 2018;27(3–4):502–15.

11. Digby R, Lee S, Williams A. The experience of people with dementia and nurses in hospital: an integrative review. J Clin Nurs. 2017;26(9–10):1152–71.

12. Digby R, Williams A, Lee S. Nurse empathy and the care of people with dementia. Australian Journal of Advanced Nursing, The. 2016;34(1):52–9.

13. Tropea J, LoGiudice D, Liew D, et al. Caring for people with dementia in hospital: findings from a survey to identify barriers and facilitators to implementing best practice dementia care. Int Psychogeriatr. 2017;29(3):467–74.

14. Royal College of Emergency Medicine. Best Practice Guidelines, Emergency Department Care [online]. 2017. http://www.rcem.ac.uk/docs/College%20Guidelines/Emergency%20Department%20Care%20-%20BP%20Guidance%20-%20Jul%202017%20-%20v6.pdf (accessed 17 March 2023).

15. Kennedy M, Koehl J, Shenvi CL, et al. The agitated older adult in the emergency department: a narrative review of common causes and management strategies. J Am Coll Emerg Physicians Open. 2020;1(5):812–23.

16. Bracken-Scally M, Keogh B, Daly L, et al. Assessing the impact of dementia inclusive environmental adjustment in the emergency department. Dementia. 2021;20(1):28–46.

17. Ricker JR, Mulligan M. Activity kits as a first line intervention to care for individuals with dementia. Geriatr Nurs (Minneap). 2017;38(6):604–5.

18. Fisher JE, Buchanan JA. Presentation of preferred stimuli as an intervention for aggression in a person with dementia. Behavior Analysis: Research and Practice. 2018;18(1):33.

19. Parke B, Hunter KF. The dementia-friendly emergency department: An innovation to reducing incompatibilities at the local level. Healthcare Management Forum. Los Angeles, CA: SAGE Publications 2017. p. 26–31.

20. Alzheimer's Society. This is me [online]. 2010. https://www.alzheimers.org.uk/sites/default/files/2020-03/this_is_me_1553.pdf (accessed 3 July 2023).

21. NHS England. A&E Attendances and Emergency Admissions 2023-24 [online]. 2023. Available from: https://www.england.nhs.uk/statistics/statistical-work-areas/ae-waiting-times-and-activity/ae-attendances-and-emergency-admissions-2023-24/ (accessed 3 July 2023).

22. North Bristol NHS Trust. North Bristol NHS Trust Dementia Strategy 2022 - 2025 [online]. 2022. https://www.nbt.nhs.uk/patients-carers/coming-hospital/dementia-care/north-bristol-nhs-trust-dementia-strategy-2022-2025 (accessed 3 July 2023).

For peer review only - http://bmjopen.bmj.com/site/about/guidelines.xhtml

23. Alzheimer's Society. Dementia: Bath and North East Somerset [online]. 2018. https://democracy.bathnes.gov.uk/documents/s50668/Dementia%20-%20Bath%20and%20North%20East%20Somerset.pdf (accessed 3 July 2023).

24. Malterud K, Siersma VD, Guassora AD. Sample size in qualitative interview studies: guided by information power. Qual Health Res. 2016;26(13):1753–60.

25. Manning SN. Managing behaviour that challenges in people with dementia in the emergency department. Emergency Nurse. 2021;29(3).

26. Braun V, Clarke V. Using thematic analysis in psychology. Qual Res Psychol. 2006;3(2):77–101.

27. Parke B, Hunter KF, Schulz ME, et al. Know me–A new person-centered approach for dementia-friendly emergency department care. Dementia. 2019;18(2):432–47.

28. Chapman R, Ogle KR, Martin C, et al. Australian nurses' perceptions of the use of manual restraint in the Emergency Department: a qualitative perspective. J Clin Nurs. 2016;25(9–10):1273–81.

29. Woods DL, Buckwalter K. Taking another look: Thoughts on behavioral symptoms in dementia and their measurement. Healthcare 2018; 6(4):126.

30. Kramer HS, Drews FA. Checking the lists: A systematic review of electronic checklist use in health care. J Biomed Inform. 2017;71:6–12.

31. Alzheimer's Society. Optimising treatment and care for people with behavioural and psychological symptoms of dementia: A Best Practice Guide for Health and Social Care Professionals [online]. 2011. https://www.alzheimers.org.uk/sites/default/files/2018-08/Optimising%20treatment%20and%20care%20-%20best%20practice%20guide.pdf?downloadID=609 (accessed 3 July 2023).

For peer review only - http://bmjopen.bmj.com/site/about/guidelines.xhtml

Dementia in the ED Survey Questions

Start of Block: Default Question Block

Welcome to 'Managing challenging behaviour related to patients in Dementia in the Emergency Department (ED)' online survey. This survey should take no longer than 5 minutes to complete. Before taking part, please read the information and consent form below, and click on the "I consent" button at the bottom of the page if you understand the information and freely consent to participate in the study.

INFORMATION AND CONSENT FORM

This survey is designed to understand ED staff experiences of challenging behaviour from patients with dementia and what interventions they have found useful. This is being led by Dr Sarah Voss and Dr Laura Goodwin of the University of the West of England, Bristol. It has been approved by the University of the West of England Research Ethics Committee.

The aim of this survey is to discover how staff in an ED department manage challenging behaviour exhibited by people with dementia in order to inform the development of a standardised de-escalation tool. These patients can often feel confused and disorientated in a hospital setting and may not be able to express pain or basic care needs. It is important they receive quality care and are as comfortable as possible. Therefore, we are interested to know why these patients may become agitated and how they are managed effectively by staff to avoid distress. The findings from this survey will be combined with data from interviews with ED staff, and may lead on to the development of an intervention to manage challenging behavior from patients with dementia in the ED.

You have been invited to take part because you are a member of staff working in the Emergency Department at either University Hospitals Bristol NHS Foundation Trust, North Bristol NHS Trust, or Royal United Hospitals Bath NHS Foundation Trust.

Participation will involve answering a series of questions about your experiences of challenging behaviour associated with dementia, whilst working in the ED. Survey participation is entirely voluntary, and there are no consequences of either choosing or not choosing to participate. You can stop being part of the study at any time, without giving a reason, but we will keep information about you that we already have. Your rights to access, change or move your information are limited, as we need to manage your information in specific ways in order for the research to be reliable and accurate. If you withdraw from the study, we will keep the information that we have already obtained. You can find out more about how we use your information by contacting dataprotection@uwe.ac.uk or by following this link:

For peer review only - http://bmjopen.bmj.com/site/about/guidelines.xhtml

www.hra.nhs.uk/information-about-patients/

Please read the following Privacy Notice before continuing.

For more information and advice, you may contact Dr Sarah Voss by phone (0117 328 8906) or email (sarah.voss@uwe.ac.uk). Alternatively you can contact Dr Laura Goodwin (laura.goodwin@uwe.ac.uk).

Q2 Do you consent to taking part in this study?

○ I consent (1)

○ I do not consent (2)

End of Block: Default Question Block

Start of Block: Block 3

Q3 Please select which job title best describes your role:

○ Nurse (1)

○ Doctor (2)

○ Porter (3)

○ Security staff (4)

○ Receptionist (5)

○ Admin staff (6)

○ Other (please state) (7) ____

For peer review only - http://bmjopen.bmj.com/site/about/guidelines.xhtml

For peer review only

Q4 How long have you worked in an Emergency Department?

◯ Less than a year (1)

◯ 1-2 years (2)

◯ 3-5 years (3)

◯ 6-10 years (4)

◯ 11-15 years (5)

◯ 16-20 years (6)

◯ 21-30 years (7)

◯ 30 years+ (8)

End of Block: Block 3

Start of Block: Block 1

Q5 Have you ever received staff training in relation to dementia?

◯ Yes (1)

◯ No (2)

Skip To: End of Block If Q5 = No

For peer review only

For peer review only - http://bmjopen.bmj.com/site/about/guidelines.xhtml

Q6 When did you receive this training? *(if you have received more than one lot of training, please indicate when you received the **most recent** training)*

- ◯ In the last year (1)
- ◯ In the last 2-5 years (2)
- ◯ 6-10 years ago (3)
- ◯ 10+ years ago (4)

Q7 Did this training involve information about how to manage challenging behaviour associated with dementia?

- ◯ Yes (1)
- ◯ No (2)

Skip To: End of Block If Q7 = No

Q8 How useful was this information?

- ◯ Extremely useful (1)
- ◯ Very useful (2)
- ◯ Moderately useful (3)
- ◯ Slightly useful (4)
- ◯ Not at all useful (5)

End of Block: Block 1

Start of Block: Block 4

For peer review only - http://bmjopen.bmj.com/site/about/guidelines.xhtml

For peer review only

UWE Bristol University of the West of England

Q9 Have you experienced challenging behaviour from patients with dementia (or suspected dementia) in the ED?

- Yes (1)
- No (2)

Skip To: End of Block If Q9 = No

For peer review only

Q10 How frequently would you say that you experience/have to manage challenging behaviour associated with dementia (or suspected dementia) in the ED?

- Daily (1)
- Weekly (2)
- Monthly (3)
- 2-3 times a year (4)
- Less than 3 times a year (5)

Q11 How many staff members are usually required to manage instances of challenging behaviour associated with dementia (or suspected dementia) in the ED?

- 1 (1)
- 2 (2)
- 3 (3)
- more than 3 (4)

For peer review only - http://bmjopen.bmj.com/site/about/guidelines.xhtml

Q12 Please tick all words which describe your experiences of challenging behaviour associated with dementia (or suspected dementia) in the ED.

- ☐ Verbal aggression (1)
- ☐ Physical assault (2)
- ☐ Agitation (3)
- ☐ Frustration (4)
- ☐ Communication difficulties (5)
- ☐ Use of swear words (6)
- ☐ Physical resistance to care (7)
- ☐ Emotional distress (8)
- ☐ Confusion (9)
- ☐ Wandering (10)
- ☐ Disorientation (11)
- ☐ Other (please state) (12) ____

For peer review only

For peer review only - http://bmjopen.bmj.com/site/about/guidelines.xhtml

Q13 How is this behaviour usually managed?

- ▢ Pharmaceutical measures (1)
- ▢ Distraction techniques (2)
- ▢ Use of family members/carers (3)
- ▢ Restraint (4)
- ▢ Moving the patient to a quieter environment (5)
- ▢ Other (please state) (6) ____

Q14 In the last three months, which of the following behaviour management practices have you observed?

- ▢ Pharmaceutical measures (1)
- ▢ Distraction techniques (2)
- ▢ Use of family members/carers (3)
- ▢ Restraint (4)
- ▢ Moving the patient to a quieter environment (5)
- ▢ Other (please state) (6) ____

End of Block: Block 4

Start of Block: Block 2

For peer review only - http://bmjopen.bmj.com/site/about/guidelines.xhtml

For peer review only

UWE Bristol University of the West of England

Q15 Which of the following do you feel are triggers for challenging behaviour associated with dementia (or suspected dementia) in the ED?

- ☐ Patient is in pain (1)
- ☐ Mental capacity of patient (2)
- ☐ Patient does not have their regular medication with them (3)
- ☐ Difficulty communicating with staff/being understood (4)
- ☐ Basic care needs are not met (5)
- ☐ Undiagnosed symptoms (6)
- ☐ ED waiting times (7)
- ☐ Noise of the ED environment (8)
- ☐ Not given enough time/attention by staff (9)
- ☐ Other (please state) (10) ____
- ☐ ⊗None of the above (11)

For peer review only

Q16 Do you feel that these instances of challenging behaviour could be **prevented**?

- ○ Yes (1)
- ○ No (2)
- ○ Sometimes (3)

Skip To: Q18 If Q16 = No

For peer review only - http://bmjopen.bmj.com/site/about/guidelines.xhtml

Q17 What do you feel could be done to **prevent** instances of challenging behaviour associated with dementia (or suspected dementia) in the ED?

- ☐ Shorter waiting times (1)
- ☐ Rapid assessment (2)
- ☐ Clearer hospital signage (3)
- ☐ Specialist input (i.e. Geriatrician) (4)
- ☐ 1:1 care from staff/carer (5)
- ☐ Written information for staff regarding patient's medical background/preferences (6)
- ☐ Quieter environment (7)
- ☐ Dimmer lights (8)
- ☐ Snacks provided (9)
- ☐ Provide distraction (i.e. music, photos, TV, puzzles, twiddle muff, other activities) (10)

For peer review only - http://bmjopen.bmj.com/site/about/guidelines.xhtml

Q18 What do you feel could be done to **de-escalate** instances of challenging behaviour associated with dementia (or suspected dementia) in the ED?

- ▢ Specialist input (i.e. Geriatrician) (1)
- ▢ 1:1 care from staff/carer (2)
- ▢ Written information for staff regarding patient's medical background/preferences (3)
- ▢ Quieter environment (4)
- ▢ Dimmer lights (5)
- ▢ Snacks provided (6)
- ▢ Provide distraction (i.e. music, photos, TV, puzzles, twiddle muff, other activities) (7)
- ▢ Pharmaceutical measures (8)

End of Block: Block 2

Start of Block: Block 5

Q19 Do you feel that there should be a separate assessment area of the ED for patients with dementia (or suspected dementia)? (please give a reason for your answer)

- ○ Yes (1) ____
- ○ No (2) ____

End of Block: Block 5

End of Survey message:

Thank you for completing this survey, your responses have been recorded.

For peer review only - http://bmjopen.bmj.com/site/about/guidelines.xhtml

If you have any questions about this project, please contact Dr Laura Goodwin (laura.goodwin@uwe.ac.uk)

For peer review only

For peer review only - http://bmjopen.bmj.com/site/about/guidelines.xhtml

Interview Topic Guide

Introductions, re-confirm consent. Explain aims of interview

Preliminary questions

Can you confirm your role within the emergency department (i.e. nurse, doctor, porter)?

How long have you worked in the ED?

Are there any dementia friendly initiatives used in your emergency department?

- What are these initiatives? How long have they been in place?

Have you received any staff training in relation to dementia?

- When? What did this entail? Did it include challenging behaviour? How useful was it?

Challenging behaviour

Have you experienced challenging behaviour from patients with dementia in the ED?

- YES: Can you tell us about these experiences?
    - What types of behaviours are exhibited?
    - How frequently do these instances occur?
    - Are these incidents reported?
    - Do you need other staff members to help? How many?
    - Did the patient need 1 to 1 care/support?
    - Are other patients impacted by this behaviour? In what way?
    - How are these situations de-escalated/ended?
    - How long do these situations last?
    - How long do these patients wait for a decision to be admitted or are they transferred to another area at the hospital such as a medical assessment unit?
    - Why do you think these situations occur? Any contributing factors/triggers?
    - Can you think of anything which may **prevent** these situations from occurring?
    - Can you think of anything which may **de-escalate** these situations once they are already happening?
- NO: If you have not had any experiences of this, why do you think these situations may occur? Any contributing factors/triggers?
    - Can you think of anything which may **prevent** these situations from occurring?
    - Can you think of anything which may **de-escalate** these situations once they are already happening?

Is there anything you can think of which could be changed about the ED environment in order to avoid challenging behaviour from patients with dementia?

- How can the organisation/staff/patients & their carers help?

For peer review only

For peer review only - http://bmjopen.bmj.com/site/about/guidelines.xhtml

Current management techniques

Have you witnessed any situations in the ED where sedation or restraint has been needed to manage challenging behaviour from patients with dementia?

Are there any assessment tools available within your ED to identify whether or not patients with dementia are likely to display challenging behaviour?

Intervention

Explain the aim to develop an intervention to prevent and/or de-escalate challenging behaviour from patients with dementia in the ED.

How do you feel about introducing this kind of intervention?

- Any possible challenges/best ways to do this?

Can you think of a way to **measure** the benefit of any new intervention?

- Physiological measures of the patient? Behavioural measures? Cognitive measures? Incident rates?

For peer review only

For peer review only - http://bmjopen.bmj.com/site/about/guidelines.xhtml

Checklist for Reporting Results of Internet E-Surveys (CHERRIES)*

Item category	Checklist item	Page no.	Description
Design	Study design	Pg 4	The target population was staff working in the emergency department of three UK Hospitals, responsible for responding to challenging behaviour. This was a convenience sample.
Ethics	Ethics approval	Pg 13	This study involves human participants and ethical approval was granted by the NHS Health Research Authority (20/HRA/0587) as well as the University of the West of England (UWE Bristol) Faculty of Health and Applied Sciences Research Ethics Committee (HAS-NAM-18-027). Participants gave informed consent to participate in the study before taking part.
	Informed consent	Pg 4	Informed consent for the survey was obtained from all those agreeing to complete a survey, with participant information displayed on the welcome page that the survey would take approximately 10 minutes to complete, that all responses were confidential and anonymous, and that data would be stored and analysed on password protected encrypted computers at UWE, accessed only by the study team, and deleted once the study findings are accepted for publication. A Privacy Notice was also available to download, and contact details were displayed for the research team. Consent was indicated when respondents clicked the 'I consent' button at the bottom of this page.<br><br>For interviews, informed consent was obtained through signed consent forms, after participants had been emailed a copy of the participant information sheet and privacy notice. Consent was then re-confirmed verbally at the start of the interview.
	Data protection	Pg 4	No personal information was collected. Survey data are stored on a secure server at UWE Bristol and the survey platform 'Qualtrics' (approved by UWE Bristol) for data security.
Development and pre-testing	Development and testing	Pg 4	The survey was designed using input from earlier research and review by the research team (healthcare professionals and researchers). A draft survey was piloted by members of the research team, as well as the patient and public involvement group.
Recruitment Process	Open vs closed Survey	Pg 4	This was a closed survey

For peer review only

	Contact mode	Pg 4	An email promoting the study (and including the survey link) was sent to all ED staff by local collaborators at each Trust and disseminated through bulletins and posters with a quick response (QR) code for the survey displayed in ED staff rooms at participating sites.
Recruitment process (cont'd)	Advertising the survey	Pg 4	As above
Survey administration	Web/email	Pg 4	This was a web-based survey, hosted by the survey platform 'Qualtrics'
	Context	Pg 4	Qualtrics is an online survey platform.
	Mandatory/voluntary	Pg 4	Voluntary.
	Incentives	N/A	N/A
	Time/date	Pg 4	Responses were collected between November 2020 and November 2021.

For peer review only

Item category	Checklist item	Page no.	Description
	Item randomisation	Appendix 1	No randomisation of items was used.
	Adaptive questioning	Appendix 1	Adaptive questioning (branched) was used. Relevant survey items were displayed based on previous responses.
	Number of items	Appendix 1	A maximum of four items were displayed on any one survey page.
	Number of screens	Appendix 1	The full survey was distributed over approximately 6 pages. A progress bar was shown at the top of the page, as the respondent was completing the survey.
	Completeness check	Appendix 1	All survey items were deemed to be mandatory, and respondents prompted to complete outstanding items before leaving the survey page on which the item was contained. Most items, except screener items and those items required for adaptive questioning included a 'Don't know/ none of the above' option.
	Review step	Appendix 1	Respondents were able to change their responses using a "Back" button at the bottom of each screen.
Response rates	Unique site visitor	N/A	N/A
	View rate	N/A	N/A
	Participation rate	N/A	N/A

For peer review only

Item category	Checklist item	Page no.	Description
	Completion rate	N/A	N/A
Preventing multiple entries from same individual	Cookies used	N/A	Not used
	IP check	N/A	Not used to maintain anonymity.
	Log file analysis	N/A	Not used.
	Registration	Pg 4	A login was not used - entry to the survey was via a web link emailed to eligible participants.
Analysis	Handling of incomplete questionnaires	Pg 4	Only completed questionnaires were included in the final dataset.
	Questionnaires with atypical timestamp	N/A	No respondents were removed from the survey for completing the items too quickly.
	Statistical correction	N/A	No weighting scheme was used for the analysis of results.

* Eysenbach G. Improving the quality of Web surveys: the Checklist for Reporting Results of Internet E-Surveys (CHERRIES). J Med Internet Res 2004;6:e34.

For peer review only

Supplementary Table 1: COREQ checklist

**Domain 1: Research team and reflexivity**		Location in manuscript (Section, page no.)
**Personal Characteristics**		
1. Interviewer/facilitator Which author/s conducted the interview or focus group?	LG, CL	Methods - 4
2. Credentials<br>What were the researcher's credentials? E.g. PhD, MD	BSc, PhD	Title Page
3. Occupation<br>What was their occupation at the time of the study?	LG: Senior Research Fellow in Emergency Care<br><br>CL: Research Associate	Title Page
4. Gender Was the researcher male or female?	Female	N/A
5. Experience and training<br>What experience or training did the researcher have?	Experienced qualitative researchers.	Methods - 4
**Relationship with participants**		
6. Relationship established<br>Was a relationship established prior to study commencement?	No	N/A
7. Participant knowledge of the interviewer<br>What did the participants know about the researcher? e.g. personal goals, reasons for doing the research	Participants were briefed on the purpose of the study and were aware that ethical approval had been granted. Participants reviewed the participant information documentation prior to giving their written informed consent to be involved.	Methods - 4
8. Interviewer characteristics What characteristics were reported about the interviewer/facilitator? e.g. Bias, assumptions, reasons and interests in the research topic	No interviewer-related biases identified.	N/A
**Domain 2: study design**		
**Theoretical framework**		
9. Methodological orientation and Theory<br>What methodological orientation was stated to underpin the study? e.g. grounded theory, discourse analysis, ethnography, phenomenology, content analysis	Thematic analysis	Methods – 5
Participant selection		
10. Sampling How were participants selected? e.g.	Convenience sampling was used: an email promoting the study (and	Methods - 4

For Peer review only

	including the survey link) was sent to all ED staff by local collaborators at each Trust and disseminated through bulletins and posters	
11. Method of approach How were participants approached? e.g. face-to-face, telephone, mail, email	As above	Methods - 4
12. Sample size How many participants were in the study?	13 interview participants	Results - 5
13. Non-participation How many people refused to participate or dropped out? Reasons?	Of the first 13 respondents that were invited for a semi-structured interview, all gave informed consent and completed the interview. There were no participants who subsequently refused to participate, withdrew consent or dropped out.	N/A
**Setting**		
14. Setting of data collection Where was the data collected? e.g. home, clinic, workplace	Data was collected via telephone and audio-recorded using Skype for business	Methods - 4
15. Presence of non-participants Was anyone else present besides the participants and researchers?	No	N/A
16. Description of sample What are the important characteristics of the sample? e.g. demographic data, date	There were mostly female (n = 10, 77%). Length of experience working in ED ranged from less than 3 years to over 10 years. Data were held between October 2020 and November 2021	Results – 5-6
**Data collection**		
17. Interview guide Were questions, prompts, guides provided by the authors? Was it pilot tested?	Interviews were semi-structured using a topic guide; the topic guide was developed and reviewed by the study team. This topic guide was then reviewed and refined by the Patient and Public Involvement contributors.	Methods - 4
18. Repeat interviews Were repeat interviews carried out? If yes, how many?	No	N/A

For peer review only

19. Audio/visual recording<br>Did the research use audio or visual recording to collect the data?	The semi-structured interviews were audio recorded using skype for business.	Methods - 4
20. Field notes<br>Were field notes made during and/or after the interview or focus group?	No additional field notes were made.	N/A
21. Duration What was the duration of the interviews or focus group?	The interviews lasted an average of 45 minutes	Methods - 4
22. Data saturation<br>Was data saturation discussed?	We used information power	Methods - 4
23. Transcripts returned Were transcripts returned to participants for comment and/or correction?	No	N/A
**Domain 3: analysis and findings**		
**Data analysis**		
24. Number of data coders<br>How many data coders coded the data?	Two – LG and CL	Methods - 5
25. Description of the coding tree<br>Did authors provide a description of the coding tree?	Coding described in methods section.	Methods - 5
26. Derivation of themes<br>Were themes identified in advance or derived from the data?	Themes were derived from the data	Methods - 5
27. Software What software, if applicable, was used to manage the data?	NVivo 12	Methods - 5
28. Participant checking<br>Did participants provide feedback on the findings?	No	N/A
**Reporting**		
29. Quotations presented Were participant quotations presented to illustrate the themes / findings? Was each quotation identified? e.g. participant number	Yes	Results – 5-11
30. Data and findings consistent<br>Was there consistency between the data presented and the findings?	Yes	N/A
31. Clarity of major themes<br>Were major themes clearly presented in the findings?	Yes	Results 5-11
32. Clarity of minor themes<br>Is there a description of diverse cases or discussion of minor themes?	Yes	Results 5-11

For peer review only
